# Precise diagnosis and typing of early-stage renal immunoglobulin-derived amyloidosis by label-free quantification of parallel reaction monitoring-based targeted proteomics

**DOI:** 10.1186/s12882-023-03105-5

**Published:** 2023-03-10

**Authors:** Yuan Li, Ying Zhang, Xinjin Zhou, Xinli Xue, Muxi Wang, Dedong Kang, Yali Zhou, Ruimin Hu, Songxia Quan, Guolan Xing, Jinghua Yang

**Affiliations:** 1 Department of Nephrology, the First Affiliated Hospital of Zhengzhou University, No. 1 East Jianshe Road, Zhengzhou, 450052 Henan China; 2 Renal Path Diagnostics at Pathologists BioMedical Laboratories, Lewisville, TX 75067 USA; 3 Clinical Systems Biology Key Laboratories of Henan, Translational Medicine Center, the First Affiliated Hospital of Zhengzhou University, No. 1 East Jianshe Road, Zhengzhou, 450052 Henan China; 4 Department of Chemistry, University of Illinois at Urbana-Champaign, Urbana, 61801 USA; 5 Department of Anatomy, Showa University School of Medicine, 1-5-8 Hatanodai, Shinagawa-Ku, Tokyo, 1428555 Japan

**Keywords:** Amyloidosis, Kidney, Targeted proteomics, Parallel reaction monitoring, Laser microdissection

## Abstract

**Background:**

Early diagnosis and typing are crucial for improving the prognosis of patients with renal amyloidosis. Currently, Untargeted proteomics based precise diagnosis and typing of amyloid deposits are crucial for guiding patient management. Although untargeted proteomics achieve ultra-high-throughput by selecting the most abundant eluting cationic peptide precursors in series for tandem MS events, it lacks in sensitivity and reproducibility, which may not be suitable for early-stage renal amyloidosis with minor damages. Here, we aimed to develop parallel reaction monitoring (PRM)-based targeted proteomics to achieve high sensitivity and specificity by determining absolute abundances and codetecting all transitions of highly repeatable peptides of preselected amyloid signature and typing proteins in identifying early-stage renal immunoglobulin-derived amyloidosis.

**Methods and results:**

In 10 discovery cohort cases, Congo red-stained FFPE slices were micro-dissected and analyzed by data-dependent acquisition-based untargeted proteomics for preselection of typing specific proteins and peptides. Further, a list of proteolytic peptides from amyloidogenic proteins and internal standard proteins were quantified by PRM-based targeted proteomics to validate performance for diagnosis and typing in 26 validation cohort cases. The diagnosis and typing effectiveness of PRM-based targeted proteomics in 10 early-stage renal amyloid cases was assessed via a comparison with untargeted proteomics. A peptide panel of amyloid signature proteins, immunoglobulin light chain and heave chain in PRM-based targeted proteomics showed significantly distinguishing ability and amyloid typing performance in patients. The diagnostic algorithm of targeted proteomics with a low amount of amyloid deposits in early-stage renal immunoglobulin-derived amyloidosis showed better performance than untargeted proteomics in amyloidosis typing.

**Conclusions:**

This study demonstrates that the utility of these prioritized peptides in PRM-based targeted proteomics ensure high sensitivity and reliability for identifying early-stage renal amyloidosis. Owing to the development and clinical application of this method, rapid acceleration of the early diagnosis, and typing of renal amyloidosis is expected.

**Supplementary Information:**

The online version contains supplementary material available at 10.1186/s12882-023-03105-5.

## Introduction

Amyloidosis is a morbid state caused by extracellular accumulation of fibrils with highly organized aggregation from several misfolded precursor proteins in an insoluble β-pleated physical conformation [1, 2]. Fibrillary structures are resistant to proteolysis, leading to the cumulative disruption of tissue structure and progressive organ dysfunction [3]. Amyloidosis can cause systemic or local lesions; kidney was the most commonly affected organ by systemic amyloidosis [4, 5]. Renal amyloidosis is a rare and intractable protein misfolding disorder which prompts progressive renal insufficiency [6]. Renal amyloidosis manifests as proteinuria, edema, and hypoalbuminemia, resulting in nephrotic syndrome via fibril deposition in the mesangium and capillary loops of the glomerulus, vessels, as well as interstitium [7, 8]. In routine practice, Renal pathology is the main basis for clinical diagnosis of renal amyloidosis. Amyloid deposits can be ascertained by typical apple-green birefringence on Congo red (CR) staining under polarized light and histopathologic ultrastructural characteristics of randomly oriented non-branching fibrils with a diameter of 7–14 nm under electron microscopy [2, 4]. Different amyloidosis types were determined according to the type of amyloid precursor protein. Serum amyloid P component (SAP), apolipoprotein E(APOE), and apolipoprotein A-IV (APOA4) universally co-deposit with precursor proteins in all types of amyloid fibrils, and these accompanying proteins are known as ‘amyloid signatures’ [1]. More than 35 amyloid precursor proteins are known as classes of heterogeneous clinical phenotypes [1]. Clinically, the most common types of amyloid fibrils in patients with renal amyloidosis are derived from fragments of immunoglobulin (Ig) lambda or kappa light-chain (AL), and rarely Ig heavy chains only (AH) or heavy chains and light chains (AHL) [9, 10]. Although most types of amyloidosis are progressive, often fatal, and manifest similarly, treatment strategies are drastically different [6, 11]. Establishing the precise nature of amyloid precursor protein is of paramount importance in guiding the clinical management of amyloidosis by reducing the production of the respective underlying etiology [5].

The incidence of AL amyloidosis is stable over the years, but a progressive decrease in delay from symptoms onset and diagnosis was reported [12]. This increased awareness may bring patients to the attention of the nephrologist even earlier than in the past, so it is necessary to lean on highly sensitive and sophisticated tools which are able to diagnose AL amyloidosis even at early stages. The renal biopsy plays a central role for the diagnosis of different forms of monoclonal gammopathy of renal significance (MGRS), helping in identifying patients with sub-detectable neoplastic clones. In this setting, proteomic techniques can play a crucial role in identifying early pathological glomerular modifications and shedding light on the pathobiology of these diseases [13]. Proteomics has been proposed the gold standard technique, with much higher sensitivity and specificity as compared to the “routine” immuno-based methods, which makes this tool necessary for the correct classification of amyloid deposits. Conventional CR histology followed by antibody-based determination and established laser microdissection/mass spectrometry (LMD/MS)-based untargeted proteomics application are sufficient for diagnosing and differentiating the types of most amyloid fibrils [11, 14, 15]. Nevertheless, data-dependent acquisition (DDA)-based untargeted proteomics may not be suitable for reliable identification and subtyping in early-stage renal amyloidosis with early lesions, such as only partially obliterated glomeruli by amyloid deposits and delicate ‘chicken-wire’ type deposits within the specimen with a CR score of 1 + deposits (on a scale of 0–3 +), which can be easily missed if no immunofluorescence (IF) or electron microscopy (EM) is performed and not amenable to laser microdissection [16–20]. As the most widespread shotgun proteomics, DDA based untargeted proteomics detected the masses of eluting cationic peptide precursors in a MS scan, and the most abundant precursors are selected in series for tandem MS (MS/MS). Although this approach certainly can achieve ultra-high-throughput, unfortunately the overlap of identifications in replicates is low (35–60%) and it lacks sensitivity and reproducibility in detecting low-abundance proteins [21]. Such global measurements generally lack precision of quantification, have inherently poor reproducibility, and overshadow deposits by other proteins due to the stochastic selection of precursor ions for MS/MS fragmentation [22]. these limitations have propelled a recent fervor in introducing parallel reaction monitoring (PRM), an acquisition method of targeted proteomics employed on an ultrahigh resolution MS [23, 24], which offers a route to determine absolute abundance and has been increasingly accepted for simultaneous determination and label-free quantification of multiple peptides for corresponding proteins due to easy development and standardization for clinical application [25]. In this study, we aimed to explore and evaluate the diagnostic performance of combining LMD and PRM-based targeted proteomics to generate rapid, highly reliable, and repeatable identification of amyloidogenic proteins for diagnosis and subtyping of renal Ig-derived amyloid deposits in a clinically applicable format.

### Patients and methods

#### Case selection

Of 46 patients evaluated in this study, 36 representative cases randomly divided into two groups, 10 cases in the discovery cohort and 26 cases in the validation cohort. 10 cases of early-stage renal amyloidosis were included in the comparison cohort. 10 cases of normal renal peritumoral tissues in total nephrectomies was normal controls. All patients underwent native renal biopsy and were diagnosed with renal amyloidosis at our institution between January 2019 and December 2020. These cases were required to meet the following criteria: CR staining was positive and apple-green birefringence was observed under polarized light; unbranched and randomly arranged fibrous at 7–14 nm under electron microscopy. The amyloid deposit types of the cases were characterized by immunofluorescence, immunohistochemistry, serum or urine test of immunofixation electrophoresis, serum or urine free light chain and Flow cytometry results of bone marrow samples. All cases were diagnosed clinically and pathologically after a review of the clinical data and standard technical tissue processing by consensus among experienced nephrologists and nephropathologists. All of 10 cases in the discovery cohort were immunoglobulin-derived renal amyloidosis. Of these, two were AL kappa cases, four were AL lambda cases, two were AHL IgG1-lambda cases, and two were AH IgG1 cases. The validation cohort comprised 26 cases, including 15 AL lambda cases, two AL kappa cases, three AH IgG1 cases, two AHL IgG1-lambda cases, one AHL IgG1-kappa case and three cases of non-immunoglobulin-derived amyloidosis. In the early-stage renal amyloidosis cohort, there were 10 cases, nine of which were AL lambda cases, and one was an AHL IgG1-lambda case. Additionally, 10 cases of normal renal peritumoral tissues from non-amyloidosis patients who underwent total nephrectomies were collected. The Ethics Committee of the First Affiliated Hospital of Zhengzhou University approved the study protocol. All methods were performed in accordance with the relevant guidelines and regulations. All patients provided written informed consent for have their medical records according to the ethics committee requirements.

#### Sample preparation

Briefly, 8-μm thick sections of formalin-fixed and paraffin-embedded renal tissue were cut onto membrane glass slides (Leica NO.11600288, Leica Microsystems, Mannheim, Germany), deparaffinized, and stained with CR. Amyloid deposit tissues were identified under fluorescent light and microdissected using laser capture techniques (Leica LMD7, Leica Microsystems, Mannheim, Germany) with a total area of approximate 50,0000 μm^2^ for heavy amyloid deposits and below 30,000 μm^2^ for early-stage amyloid deposits per sample. Normal control tissues were microdissected and collected with the same area of glomerulus and tubulointerstitium in total of approximate 50,0000 μm^2^. Microdissected tissues were collected into PCR tube caps containing 20 µL Tris/EDTA/0.002% Zwittergent 3–16 buffer.

#### DDA analysis

Samples were heated for de-crosslinking and then ultrasonicated. The proteins extracted from the microdissected tissue fragments were digested into proteolytic peptides using 0.2 μg trypsin (Promega Corporation, Madison, WI) overnight at 37 °C. After desalting using ZipTip C18 micro columns (Millipore, Billerica, MA), samples were analyzed using an EASY-nLC 1200 liquid chromatography system with Reprosil-Pur 120 C18 columns (150 μm * 25 cm, 1.9 μm) in online separation coupled to a Q-Exactive HF-X Orbitrap mass spectrometer (Thermo Fisher Scientific GmbH, Bremen, Germany) using a mobile phase consisting of buffer A (0.1% formic acid, FA) and a 120-min gradient of increasing concentrations of buffer B (0.1% FA, 80% acetonitrile) at a flow rate of 600 nL per minute. The linear gradient method began at 4% buffer B, and the concentration was increased to 7% at 1 min, with subsequent increases to 25% (95 min), 40% (111 min), 100% (116 min), and a hold for 4 min. Mass spectra were acquired under data-dependent acquisition (DDA) mode, consisting of full MS1 scans (m/z range, 350–2000; resolution: 60,000) followed by MS2 scans of the top 20 parent ions (resolution of 15,000 AGC target to 5e^4^, maximum injection time of 45 ms).

#### Data acquisition

MS raw data were queried with the Sequest search engine against human entries in the UniProtKB/SwissProt Human Protein database concatenated with reverse decoy database in Proteome Discoverer software (version 2.4, Thermo Fisher Scientific GmbH, Bremen, Germany). The following search parameters were used: the cleavage enzyme, trypsin; precursor mass tolerance, 10 ppm; and fragment mass tandem tolerance, 0.02 Da, allowing up to three missed cleavages and variable modifications: methionine oxidation. Protein identifications were accepted if they were identified with high confidence (false discovery rate FDR < 0.01). Potential contaminant proteins were identified and removed from the search results. Protein abundances were calculated by the abundance of all matched peptides that were not shared between different proteins or protein groups and shared peptides used for the protein that had more identified peptides only. For each sample, normalization of protein abundance was processed with the algorithm based on the total sum of all protein abundance normalized to constant values of 10^6^. The protein abundance ratio of renal amyloidosis samples to normal controls was directly calculated.

#### PRM analysis

PRM mode was developed to verify and quantify the relative abundance of candidate proteins. Justification for targeted peptides was selecting non-redundant precursor peptides (7–25 amino acids in length) of each targeted protein with ranking of peptide intensities based on the global DDA mode, and filtering through a set of criteria included minimizing miscleavage sites, constrained with characters of + 2 or + 3 charge, the number of variable modifications, and C-terminal cysteine or glutamine as well as methionine when possible.

Sample preparation for PRM methods in 36 specimen sets (26 renal amyloid deposit tissues vs. 10 normal controls) was the same as that of the DDA mode described previously. Samples were analyzed using an EASY-nLC 1200 liquid chromatography system with PepMap C18 columns (75 μm*15 cm, 2 μm) coupled to a Q-Exactive HF-X Orbitrap mass spectrometer (Thermo Fisher Scientific GmbH, Bremen, Germany) using a mobile phase consisting of buffer A (0.1% FA) and a 30-min linear gradient of increasing concentrations of buffer B (0.1% FA, 80% acetonitrile) at a flow rate of 500 nL/min. The linear gradient method began at 6% buffer B, and the concentration was increased to 25% at 21 min, with subsequent increases to 40% (25 min) and 100% (28 min), and then a hold for 2 min. Mass spectra were acquired using the PRM mode, consisting of full MS1 scans and targeted MS2 scans of the top 20 parent ions (resolution at 30,000, AGC target to 2e^5^, maximum injection time of 70 ms). The retention time windows in Tables 2 and 3 were used to schedule the targeted peptide acquisition in the PRM method.

#### Quantitative analysis

Skyline software (version 20.1, MacCoss Lab Software) was used to process the PRM data [26]. The intense product ion peaks for each precursor in the extracted ion chromatograms (XICs) corresponding to each peptide were selected from the MS2 spectra based on the retention time between samples. Several internal standard candidate proteins and corresponding peptides were screened and optimized based on weighted sum of full MS1 scans for relative quantitation. The relative concentration abundance was expressed as the sum of the area under the curve (AUC) of all product fragment ion peaks of proteolytic peptides and peak area ratio to that of internal standard peptides. Receiver operating characteristic (ROC) analysis was performed using the R package, pROC [27]. ROC analysis was performed for renal amyloidosis against controls using the abundance ratio values for peptides analyzed using PRM for each selected protein. Logistic regression models were applied to assess the diagnostic quality of the combination of separate peptides for each protein in renal amyloidosis. The peptide abundance ratios of the amyloidogenic protein in controls were measured by the same methods using nephrectomy specimens from patients with renal peritumoral renal tissues in total nephrectomies lacking renal parenchymal abnormality. Cut-off values for diagnosis and typing of early-stage renal amyloidosis by PRM based targeted proteomics were referenced through maximal peptide abundance ratios of amyloidogenic protein in control tissues.

#### Statistical analysis

Statistical evaluation was conducted using Excel (Microsoft Corporation, NY, USA), SPSS version 21.0 software (SPSS Inc., Chicago, IL, USA), and R programming language version 3.5.1 (R Foundation for Statistical Computing, https://www.r-project.org/). Comparison between two groups of normal distribution variables was conducted using Student’s t-test; otherwise, the Kruskal–Wallis test was used. Statistical significance was set at *p* < 0.05.

## Results

### Baseline characteristics of renal amyloidosis patients

A cohort of 36 representative CR-positive and 10 early-stage renal amyloidosis cases were proceeded with comprehensive estimation of clinical presentation and clinical biochemistry. The clinical characteristics of patients in each group are summarized in Table 1. The median age at the time of kidney biopsy for the entire group was 59.5 years; no significant difference between the groups. The disease occurred more often in male patients, reflecting the sex imbalance observed in renal amyloidosis. Serum creatinine and 24 h urinary protein levels at biopsy were lower in patients with early-stage renal amyloidosis than those with renal amyloidosis; however, there was no significant difference in serum albumin levels. Nephrotic syndrome was present in 69.6% (32/46) of patients, and early-stage renal amyloidosis patients had a lower frequency of full nephrotic syndrome (30% vs. 80.6%, *p* = 0.002) than patients with representative renal amyloidosis. There was no significant difference in the frequency of peripheral edema between the two groups.

**Table 1:** Baseline clinical characteristics of renal amyloidosis patients

Clinical Characteristics	All Patients (*n* = 46)	Representative AL/AH/AHL Renal Amyloidosis (*n* = 36)	Early-Stage Renal Amyloidosis (*n* = 10)
Age (year)	59.5(52.25–65.75)	60(52.75–66)	55(51–63.25)
Sex (male/ female)	31/15	27/9	4/6
Serum creatinine (mg/dl)	0.85(0.71–1.37)	0.88(0.75–1.52)	0.74(0.64–0.85)
Serum albumin (g/l)	25.95(22.63–28.60)	24.45(21.70–28.60)	28.25(26.88–29.23)
24-h urine protein	4.52(2.73–6.36)	5.26(3.85–7.98)	2.15(1.52–3.16)
Peripheral edema	40	31	9
Full nephrotic syndrome	32	29	3

Data are expressed as median (interquartile range) or n. AL/AH/AHL, light chain/heavy chain/heavy and light chain amyloidosis

### Preselection of typing specific proteins and proteolytic peptides in amyloid deposits

A total of 10 representative biopsy-proven CR-positive renal amyloid deposit cases and 10 normal controls as a discovery cohort were assessed and microdissected for untargeted proteomic analysis. A total of 2819 ± 132 (mean ± SD) proteins were identified from microdissected amyloid deposits per case, and the number of proteins quantified among cases was similar. As expected, some inter-patient variations were found in the specific proteins present in deposits (particularly in proteins with low abundance), resulting in the identification of a total of 3280 proteins across all cases. Untargeted proteomic data of these proteins with significantly elevated abundance in renal biopsy amyloid deposit tissues are shown in Supplemental Table S1. High abundance ratios to control tissues in the discovery untargeted proteomic profiles discriminate hemal contaminants and structure proteins contained in amyloid deposit tissues to reduce unwanted interference during amyloid detection. The amyloid signature proteins, apolipoprotein E, serum amyloid P-component, and apolipoprotein A-IV proteins [1, 28, 29] were integrated as common constituents of amyloid deposits with high abundance in all cases. The most abundant fibril precursor protein in the untargeted proteomic data was considered for typing renal amyloidosis, such as constant and/or variable regions of Ig lambda or kappa light-chain without abundant heavy-chain regions in AL. Significant abundance elevation for Ig heavy chain regions without/with light chain regions was detected consistent with AH/AHL. From the total protein profiles identified in the 10 samples, six potential internal standard candidate proteins with easy detectability and stable expression among renal amyloidosis samples and controls were screened for relative quantitation in PRM-based target proteomic arrays (Supplemental Table S2). Initially, after step-by-step reduction in PRM analytic procedures, prioritized unique proteolytic peptides and representative transitions list per candidate protein in previous discovery proteomics phase were involved in the development of PRM analysis in renal Ig-derived amyloidosis. A list of 63 proteolytic peptides (12 candidate proteins) and product fragment ion peaks derived from the precursor ions were generated (Supplemental Table S3).

### Validation of the candidate proteins in renal amyloid deposits using PRM

**Fig. 1:**
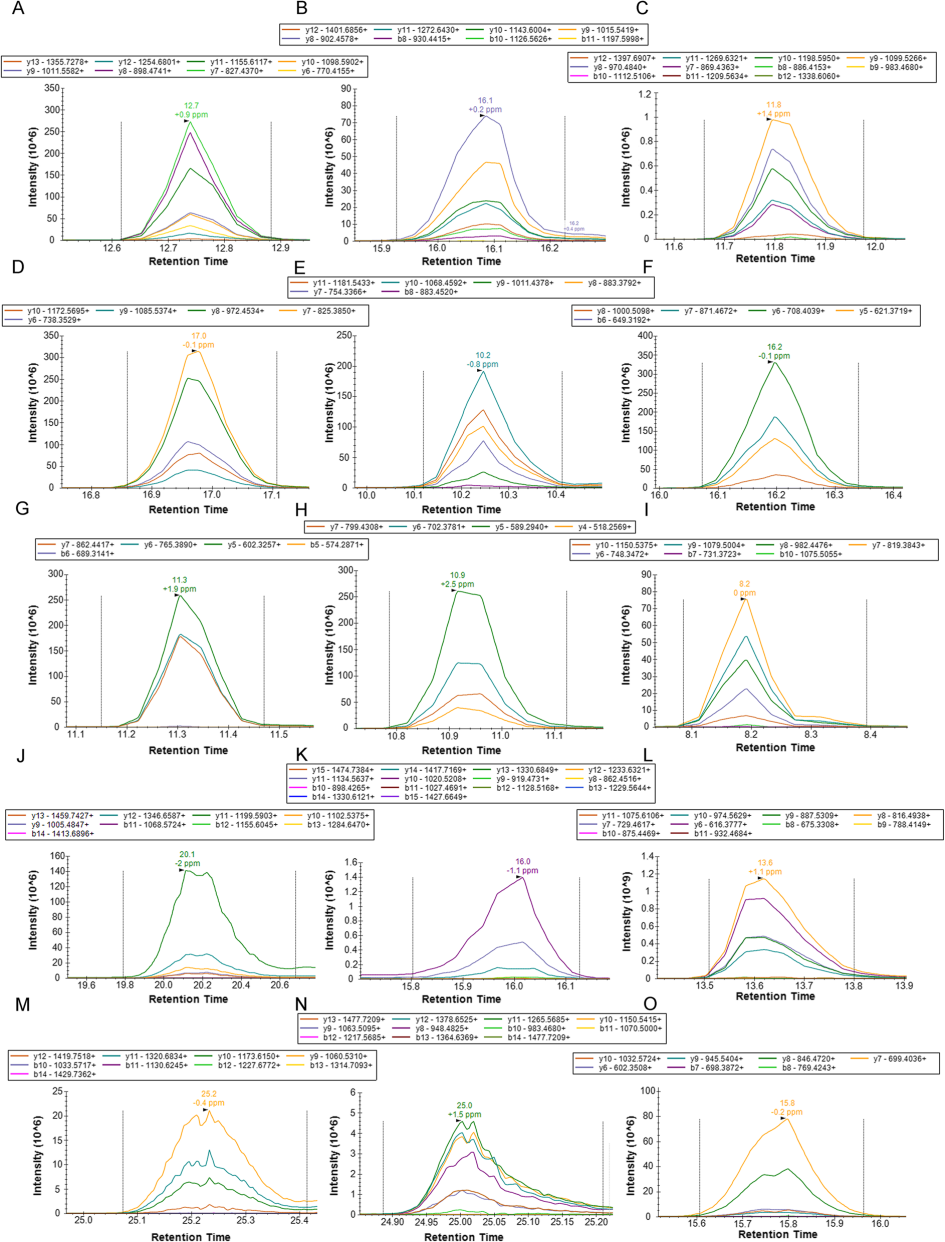
Representative extracted Ion chromatograms of targeted peptides for monitoring the expression levels of the amyloidogenic protein in parallel reaction monitoring arrays. **A**-**C** unique peptides of Apolipoprotein E: AATVGSLAGQPLQER, SELEEQLTPVAEETR, and VEQAVETEPEPELR. **D**-**F** unique peptides of Serum amyloid P-component: AYSLFSYNTQGR, IVLGQEQDSYGGK, and VGEYSLYIGR. **G**-**I** unique peptides of Apolipoprotein A-IV: LAPLAEDVR, LEPYADQLR, and SLAPYAQDTQEK. **J**-**L** unique peptides of Ig lambda light chain: AAPSVTLFPPSSEELQANK, ADGSPVNTGVETTTPSK, and SGTSASLAISGLR. **M** unique peptide of Ig kappa light chain: TVAAPSVFIFPPSDEQLK. **N**, **O** unique peptides of Ig gamma-1 heavy chain: GPSVFPLAPSSK and TTPPVLDSDGSFFLYSK

To enable outstanding performance of relative label-free quantification of multiple candidate proteins for renal amyloidosis diagnosis and typing, label-free PRM approach-based targeted proteomics are employed. Microdissected tissues from 26 renal amyloidosis patients and 10 normal controls were analyzed, and a final panel of 21 peptides belonging to 10 proteins was ultimately monitored to quantify, with high specificity, the soluble fraction of amyloid deposits (Tables 2 and 3). XICs in elution profiles showed that the peaks of peptides from the corresponding protein displayed a good response and positive spectral matching with the library in the targeted approaches (Fig. 1 and Supplemental Fig.S1). The availability of XICs in PRM acquisition guarantees accurate and fast detection of peptides in complex clinical samples. The targeted peptide expression stability of each internal standard protein was determined based on the abundance coefficient of variation (CV%) of the corresponding unique peptide cross samples following normalization to total MS1 intensity in the full scan stage. The most stable expression of selected peptide cross samples for amyloid deposits belonging to prelamin-A/C, vimentin, vinculin, and histone H4 (average CV%: 30.13%) were prioritized as an internal standard panel to quantify relatively cross renal amyloid deposits to ensure high reproducibility of the full process.

**Table 2:** Verification of PRM the sensitivity and specificity, area under the curve, and the combined effect to assess the diagnostic abilities of peptides and peptide combinations derived from amyloidogenic proteins to type renal amyloidosis

Protein Name	Peptide Sequence	Charge	Precursor Ions m/z	Retention Time Window [min]	Peptide AUC	Protein AUC
Apolipoprotein E	AATVGSLAGQPLQER	+ +	749.404629	10–17	0.8653	0.9733
Apolipoprotein E	SELEEQLTPVAEETR	+ +	865.925788	13.2–20.3	0.8584	
Apolipoprotein E	VEQAVETEPEPELR	+ +	813.404491	8.9–15.9	0.828	
Serum amyloid P-component	AYSLFSYNTQGR	+ +	703.83859	14.2–21.2	0.8109	0.9815
Serum amyloid P-component	IVLGQEQDSYGGK	+ +	697.351531	7.2–14.2	0.8182	
Serum amyloid P-component	VGEYSLYIGR	+ +	578.803488	13.3–20.3	0.8174	
Apolipoprotein A-IV	LAPLAEDVR	+ +	492.279649	8.2–15.2	0.79	0.9339
Apolipoprotein A-IV	LEPYADQLR	+ +	552.787838	8.5–15.5	0.7681	
Apolipoprotein A-IV	SLAPYAQDTQEK	+ +	675.830431	5.1–12.1	0.815	
Immunoglobulin lambda light chain	AAPSVTLFPPSSEELQANK	+ +	993.512562	17.3–24.3	0.8524	0.9227
Immunoglobulin lambda light chain	ADGSPVNTGVETTTPSK	+ +	830.904856	14–18	0.6502	
Immunoglobulin lambda light chain	SGTSASLAISGLR	+ +	610.335684	8–12	0.7214	
Immunoglobulin kappa light chain	TVAAPSVFIFPPSDEQLK	+ +	973.517116	22–29	0.8926	0.8926
Immunoglobulin gamma-1 heavy chain	GPSVFPLAPSSK	+ +	593.826963	13–20	0.8546	0.8621
Immunoglobulin gamma-1 heavy chain	TTPPVLDSDGSFFLYSK	+ +	937.464549	21.9–28.9	0.7445	

*AUC* Area under the curve

**Table 3:** the most stable 4 internal standard proteins and corresponding peptides validated by LMD/PRM-MS based targeted proteomics

Protein Name	Peptide Sequence	Charge	Precursor Ions m/z	Retention Time Window [min]	CV%
Histone H4	DNIQGITKPAIR	+ +	663.380425	6.9–13.9	20.19
Histone H4	ISGLIYEETR	+ +	590.814053	10.7–17.7	27.98
Prelamin-A/C	NSNLVGAAHEELQQSR	+ +	876.934813	7.7–14.7	33.08
Vimentin	TYSLGSALRPSTSR	+ +	748.396804	8.1–15.1	29.23
Vinculin	AVAGNISDPGLQK	+ +	635.343509	6.3–13.3	37.32
Vinculin	SLGEISALTSK	+ +	553.308603	11.8–18.8	32.95

*CV%* Coefficient of variation

### Distinguishing renal amyloid deposits by relative label-free quantification of amyloidogenic proteins using PRM analysis

**Fig. 2:**
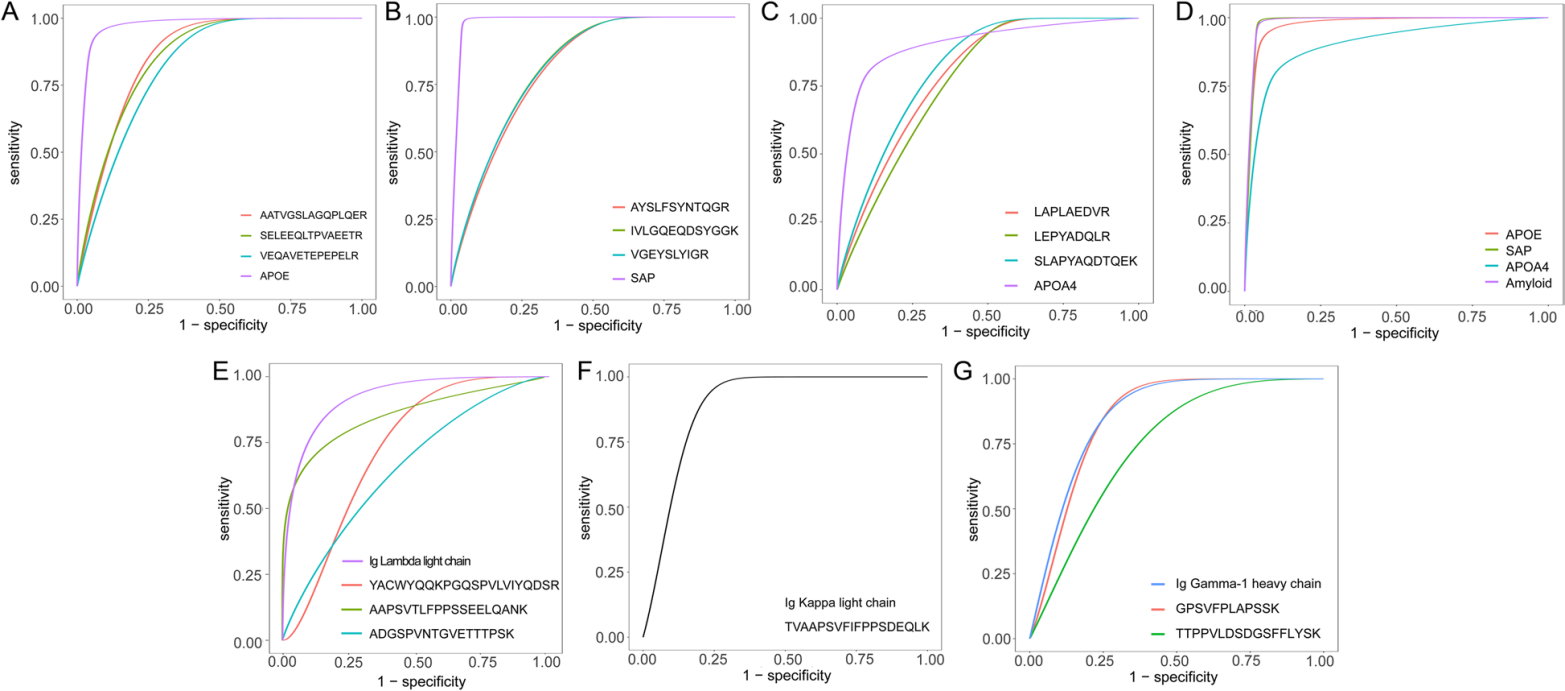
ROC curve analysis of amyloid signature and typing protein that best classify renal amyloidosis. ROC curve analysis showing the area under the curve and the combined effect to assess the diagnostic abilities of each selected peptides and peptide combinations derived from amyloid signature and typing proteins by logistic regression analysis. ROC curves for the top performing proteins that predict the classification of cases were plotted according to the ratio to internal standard proteins by LC-PRM/MS. **A**-**G** ROC curve showing the discriminatory ability of peptides for Apolipoprotein E, Serum amyloid P-component, Apolipoprotein A-IV, Ig lambda light chain, Ig kappa light chain, and Ig gamma-1 heavy chain

Diagnostic and typing models were developed by evaluating the abundance ratio of targeted amyloid signature and fibril precursor proteins, allowing a comprehensive abundance of internal standard protein panels to discriminate renal amyloid tissues**.** In the validation set, LMD/PRM-MS was successful at identifying the amyloid type in 26 (100%) of the representative cases by observing relative abundance levels higher than the cut-off value as LMD/DDA-MS and indicating the excellent specificity and cross-sample reproducibility of these candidate markers. When both methods were informative, there was 100% concordance between LMD/PRM-MS and LMD/DDA-MS. ROC analysis of the relative quantification of selected peptides revealed clinical diagnostic values that clearly demarcated patients with renal amyloidosis from controls (Fig. 2). Furthermore, the combined ROC curve of a panel including APOE, SAP, and APOA4 using logistic regression showed a high diagnostic accuracy relative to the individual proteins (Table 2). The ability of these selected amyloid precursor proteins and corresponding peptides provides an AUC of more than 0.839 as effective classifiers for distinguishing different types of renal amyloid deposits. The selected peptides of each protein also showed good complementation and correlation with relatively high AUC values in the PRM analysis validation.

### Advantages of targeted proteomics in diagnosing and typing early-stage renal amyloidosis

During the study period, 10 early-stage renal Ig-derived amyloid specimens were available to validate whether relative quantification of amyloidogenic proteins by targeted proteomics can enhance performance for differentiating typing of amyloid compared with untargeted proteomics. Generally, the diagnosis and typing of these Ig renal amyloid specimens followed standard clinical and pathologic criteria. Only five cases met the criteria for Ig amyloidosis typed by untargeted proteomics (Supplemental Table S4); however, using targeted proteomics, nine Ig amyloidosis cases were diagnosed. One Ig amyloidosis could not be diagnosed by either method owing to the low abundance of proteins/peptides for amyloid deposits. The abundance ratio of targeted peptides greater than the cut-off value was marked, and the most abundant amyloidogenic protein identified in all replicates determined the subtype. For example, Cases 1 and 2 are subtyped as AL-lambda and AHL, respectively (Table 4). Detailed targeted proteomic results are organized, and different targeted peptides of proteins with abundance ratios to internal standards are listed in Supplemental Table S5. These data suggest that targeted proteomics is more sensitive than untargeted proteomics and can detect renal amyloidosis in specimens with low amounts of amyloid.

**Table 4:** Examples from two early-stage renal amyloidosis patients

Protein Name	Peptide Sequence	CASE1-1	CASE1-2	CASE1-3	CASE2-1	CASE2-2	CASE2-3	Cut Off Value
		Abundance Ratio to internal standards						
Apolipoprotein E	AATVGSLAGQPLQER	0.5105^a^	0.3070	0.2868	2.2715^a^	2.7588^a^	2.894^a^	0.4639
Apolipoprotein E	SELEEQLTPVAEETR	0.1317^a^	0.1078^a^	0.0898 ^a^	1.1896^a^	2.2193^a^	2.3639^a^	0.0811
Apolipoprotein E	VEQAVETEPEPELR	0.0003	0.0004	0.0004	0.0075^a^	0.0254^a^	0.0246^a^	0.0012
Serum amyloid P-component	AYSLFSYNTQGR	0.4399	0.024	0.1923	0.2478	0.2921	1.7758^a^	0.7298
Serum amyloid P-component	IVLGQEQDSYGGK	2.153^a^	1.204^a^	1.0699 ^a^	2.5133^a^	4.3202^a^	4.3198^a^	0.3733
Serum amyloid P-component	VGEYSLYIGR	4.1456 ^a^	1.5855^a^	1.4676 ^a^	4.6935^a^	1.7229^a^	1.7285^a^	0.5511
Apolipoprotein A-IV	LAPLAEDVR	0.5473	0.3128	0.3027	0.2363	0.2302	0.2354	0.6155
Apolipoprotein A-IV	LEPYADQLR	0.4272	0.3423	0.3019	0.236	0.1971	0.2158	0.4947
Apolipoprotein A-IV	SLAPYAQDTQEK	0.236	0.1641	0.1453	0.1116	0.1156	0.1252	0.248
Immunoglobulin lambda light chain	AAPSVTLFPPSSEELQANK	0.6991^a^	0.4601 ^a^	0.4226	7.7564^a^	7.494^a^	9.2687^a^	0.4594
Immunoglobulin lambda light chain	AGVETTTPSK	0.1584^a^	0.1606^a^	0.152 ^a^	2.2451^a^	3.0237^a^	3.2771^a^	0.0242
Immunoglobulin lambda light chain	SGTSASLAISGLR	0.0084	0.0031	0.0026	0.0245	0.0139	0.0138	0.2104
Immunoglobulin kappa light chain	TVAAPSVFIFPPSDEQLK	0.0445	0.0279	0.0265	0.0684	0.0554	0.0818	0.7567
Immunoglobulin gamma-1 heavy chain	GPSVFPLAPSSK	0.0967	0.0744	0.0712	2.7987^a^	3.293^a^	3.7512^a^	1.0779
Immunoglobulin gamma-1 heavy chain	TTPPVLDSDGSFFLYSK	0.0087	0.0058	0.0054	0.3865^a^	0.2348^a^	0.1486	0.1543

The table displays the abundance ratio to internal standards of targeted peptide per protein corresponding the cut-off value for each patient (repeated three times). In general, the greater the abundance ratio than the cut-off value, the more significant the amyloidogenic proteins

^a^Abundance ratio of the peptide greater than the cut off value

## Discussion

Early diagnosis and precise typing amyloid fibrils is a key diagnostic procedure for better understanding of pathogenesis for rational and effective treatment strategies in renal amyloidosis. When the distribution amyloid fibril in the paraffin slices is heterogeneous or the amount of amyloid deposits in the tissue is small, it can lead to missed diagnosis or typing [4]. The diagnosis and typing of renal amyloidosis is occasionally neglected by depending only on antibody based methods (IF or IHC) [14]. Changed or deleted conformation in Ig light or heavy chain during amyloid formation affects the performance of commercially available antibodies to recognize epitopes in amyloid fibers. Contaminations by serum proteins or nonspecific charge interaction between the amyloid deposits and the reagent antibody can lead to false-positive staining [4, 5, 30]. Proteomics aims to identify all protein information in samples such as cells or tissues [6, 19]. FFPE-based proteomics is unbiased to identify all amyloid fiber protein types in a single assay, unlike immunologic-based techniques that a single test would only identify a single amyloid fibril protein type [9, 30]. With continuously developing of high-resolution mass spectrometry technology, proteomics has become a powerful tool for the identification of fibroid protein. Two distinct approaches to liquid chromatography MS/MS-based proteomics include the untargeted mode commonly employed in the global identification of proteins and the serendipitous discovery of unsuspected proteins/biomarkers [31, 32], and the targeted mode that attempts to precisely identify and quantify specific peptides of corresponding proteins [33]. Untargeted proteomic analysis is usually accomplished in a model of label-free DDA-MS, in which tandem mass spectra of several top most intense precursor ions are collected and matched with the fragmentation pattern as fingerprint characteristics of peptides corresponding to proteins by search engines with the amino acid sequences publicly available in the UniProtKB/Swiss-Prot database [29, 30]. Over the past decade, untargeted proteomic analysis of CR-positive tissues obtained by microdissection has been relatively advanced and become one of the gold standards for the identification of amyloid deposits from native kidney biopsies [4, 9, 15, 19, 28, 29], which now is routinely used in our center. Amyloid subtyping is assigned based on amyloidogenic proteins with the highest abundance and ratio detected from a protein list ranking by semi-quantitative abundance and confidence (total counts of MS/MS spectral matches and probability score) of all microdissections [15, 28].

Despite technological advances in untargeted proteomics and bioinformatics that contribute to the clinical application of diagnosis and typing of renal amyloidosis, there still are several limitations which elicit requirements for development of LMD/PRM-MS-based target approaches to diagnosis and type amyloid fibrils, such as the difficulty in detecting low-abundance proteins [19, 29]. In the context of capturing inadequate areas and quantities of amyloid tissues by LMD, such as poor smears or lack of prominent amyloid deposit, it is quite arduous as challenges in identifying the major molecular components present in renal amyloid deposits by DDA mode-based discovery untargeted proteomics owing to increased competition from tissue contaminations for the instrument time [19, 29, 34]. Thus, the factors including partial obliteration of amyloid deposition within the early stage renal amyloidosis specimen with a CR score of 1 + deposits (on a scale of 0–3 +) [20] may not be amenable to LMD as constituent proteins are overshadowed by other proteins from the background impurities in untargeted proteomic analysis.

To date, the advantages of PRM have not been evaluated or have not been commonly introduced as a clinical diagnostic technique for renal amyloidosis, especially in the early stages. In contrast to the unsupervised mode (data-dependent and data-independent acquisition), targeted MS-based approaches showed markedly better performance for easy applicability in routine clinical context with less-expensive instruments by less-experienced operators, analytical sensitivity, precision, analytical standardization, and multiplexing in the areas of tissue-based quantitative diagnostics [25, 35]. Among these, a hypothesis-driven approach, PRM, which combines the high resolving power and mass accuracy of the quadrupole-orbitrap analyzer, systematically and precisely quantifies large sets of more than the typical 500 peptides in predefined m/z ranges and retention time windows per analysis to obtain excellent and easily readable chromatogram profiles of transitions extracted from the MS/MS in complex tissues [36–38]. The PRM mode overcomes the bias toward most abundant proteins commonly observed with untargeted proteomics and represents a more efficient approach than selected reaction monitoring and multiple reaction monitoring acquisition mode in distinguishing microdissected amyloid deposit tissues in targeted proteomics owing to the systematic removal of interferences in similar composition samples by high resolution/accurate mass (HR/AM) analysis [24, 36, 39].

Based on these advantages of matching and quantifying product ions from several preselecting unique target peptides for each amyloidogenic protein, the PRM mode is faster and more precise than the DDA mode. Another strength of PRM analysis in our diagnostic algorithm is application of internal standard proteins instead of the stable isotope-labeled standard for the relative quantification of target proteins in tissue. This aspect will not only ameliorate the discrimination of tissue amount among cases, but should be advantageous and economically profitable in clinical scenarios. Accurate relative quantitation of LMD/PRM-MS partly offsets the weak enrichment of the dissected sample and decreases interference in complex backgrounds. For clinical implementation, all samples can run in standardized LMD/PRM-MS flows and abundance ratios to internal standards are determined for the diagnostic algorithm.

In this study, the diagnosis and typing effectiveness of LMD/PRM-MS was evaluated only in cases of some common renal amyloid types (AL lambda, AL kappa and AH/AHL). Non-immunoglobulin derived types, such as AA, AGel, ALys, ALECT2, Aβ2M, AFib, AApoAI, AApoAII, AApoCII, and AApoCIII, were not included due to their low incidence in these amyloidosis. The same control cases were used to assess the diagnostic specificity in both the discovery and validation sets, which have been inadequate. To affirm the high sensitivity and specificity of LMD/PRM-MS in typing renal amyloidosis, it is desirable to include more non-immunoglobulin derived amyloidosis cases and non-amyloidosis control cases in the validation sets. However, we failed to collect more cases in these respects, which are limitations of the present study. Therefore, a larger, independent, longitudinal and prospective clinical study is necessary to further optimize the LMD/PRM-MS in terms of diagnostic sensitivity and specificity. Meanwhile, the presence of abnormal biosynthesis of Ig fragments and/or post-translational proteolysis suggests that the absence of known peptides from previous data leads to false negative identification of fibril precursor proteins, particularly in the localized forms. LMD/PRM-MS is not the only solution to this issue, because relatively new in situ proteomics techniques, MALDI-MSI can also detect and type even small amount of amyloid deposits directly on one single tissue slide without the need of LMD [13, 40, 41].

In conclusion, this study highlights the utility of LMD/PRM-MS-based targeted proteomics to determine Ig-derived renal amyloidosis. Targeted proteomics overcomes some of the disadvantages of presented by untargeted proteomics, which is currently used to identify early-stage amyloid deposits, by combining sensitive and stable analytical performances. The establishment of this method promises to rapidly accelerate the development and deployment of PRM-based targeted proteomics for diagnosing and typing renal amyloidosis.

## Supplementary Information

**Additional file 1: Supplemental Tables and Figure. Supplemental Figure 1.** Representative extracted ion chromatograms of the targeted peptides of internal standard proteins. **Supplemental Table 1. **List of significantly high normalized abundance proteins in 10 discovery cohort cases and 10 controls microdissected from renal tissues in parallel. **Supplemental Table 2.** List of potential internal standard proteins identified in 10 discovery cohort cases by high normalized protein abundance and non-significant abundance ratio to 10 controls from microdissected renal tissues. **Supplemental Table 3.** List of all identified peptides of amyloidogenic proteins and internal standard proteins in 10 discovery cohort cases and 10 controls microdissected from renal tissues in parallel. **Supplemental Table 4.** Untargeted proteomic diagnostic signature for 10 cases of early-stage renal amyloidosis. **Supplemental Table 5.** PRM-based targeted proteomic diagnostic signature for 10 cases early-stage renal amyloidosis.

## Data Availability

Proteomics data generated and analysed during the current study have been uploaded in PRIDE with the dataset identifier PXD038265.

## Abbreviations

AH: Immunoglobulin heavy chain amyloidosis
AHL: Immunoglobulin heavy light chain amyloidosis
AL: Immunoglobulin light chain amyloidosis
AUC: Area under the curve
CR: Congo red
DDA: Data-dependent acquisition
FFPE: Formalin fixed paraffin-embedded
HR/AM: High resolution/accurate mass
Ig: Immunoglobulin
LMD: Laser microdissection
MS: Mass spectrometry
PRM: Parallel reaction monitoring
ROC: Receiver operating characteristic
