# Supplementary material for: Precise diagnosis and typing of early-stage renal immunoglobulin-derived amyloidosis by label-free quantification of parallel reaction monitoring-based targeted proteomics
Source: BMC Nephrol. 2023 Mar 10;24:50. doi: 10.1186/s12882-023-03105-5 (PMC9999574; doi:10.1186/s12882-023-03105-5)
Supplement: Supplementary file 1 — Additional file 1: Supplemental Tables and Figure. Supplemental Figure 1. Representative extracted ion chromatograms of the targeted peptides of internal standard proteins. Supplemental Table 1. List of significantly high normalized abundance proteins in 10 discovery cohort cases and 10 controls microdissected from renal tissues in parallel. Supplemental Table 2. List of potential internal standard proteins identified in 10 discovery cohort cases by high normalized protein abundance and non-significant abundance ratio to 10 controls from microdissected renal tissues. Supplemental Table 3. List of all identified peptides of amyloidogenic proteins and internal standard proteins in 10 discovery cohort cases and 10 controls microdissected from renal tissues in parallel. Supplemental Table 4. Untargeted proteomic diagnostic signature for 10 cases of early-stage renal amyloidosis. Supplemental Table 5. PRM-based targeted proteomic diagnostic signature for 10 cases early-stage renal amyloidosis. [file 12882_2023_3105_MOESM1_ESM.pdf]

Supplementary Material

- Supplemental Figure 1
- Supplemental Table 1-5

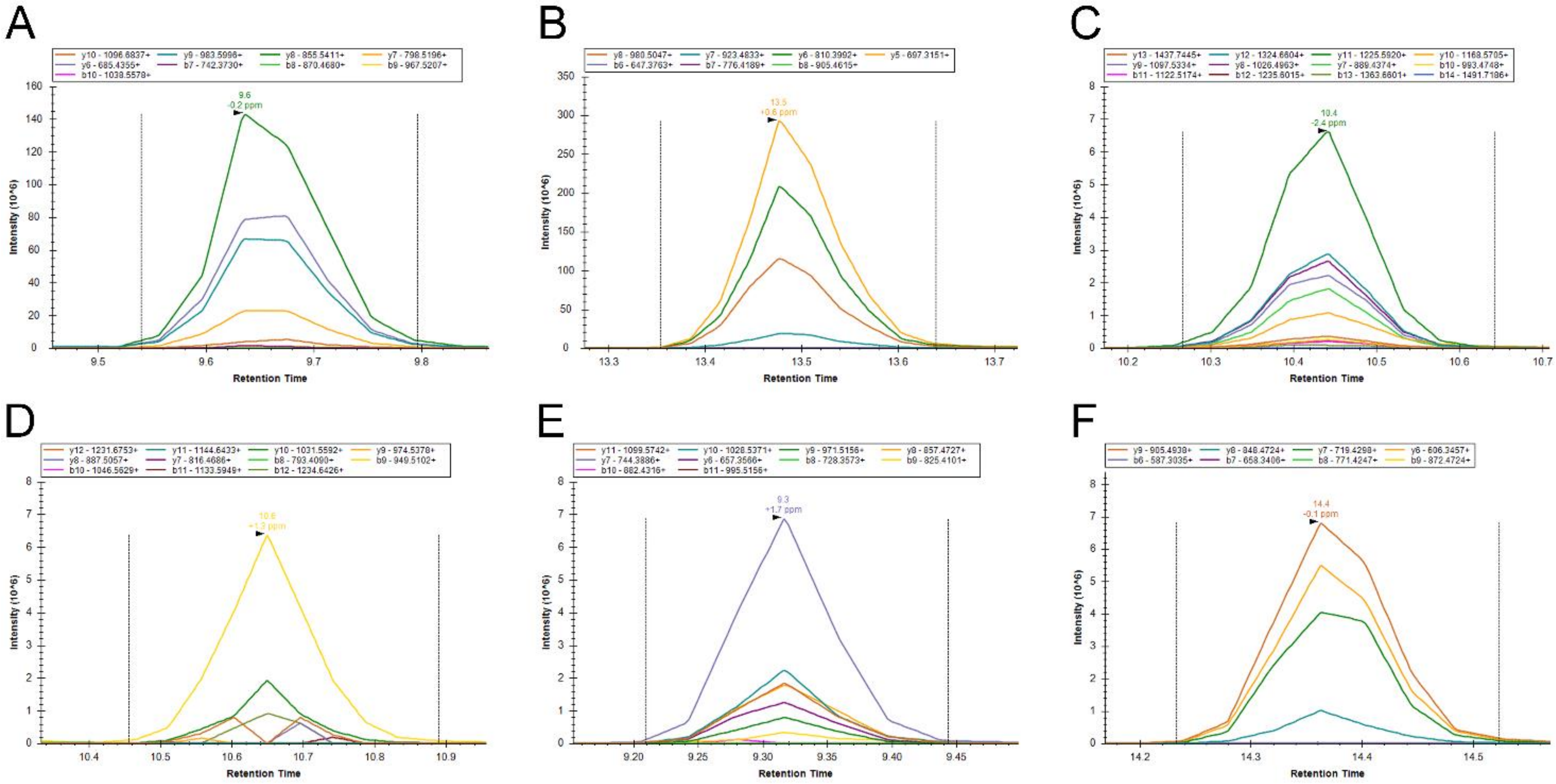

Supplemental Figure 1. Representative extracted ion chromatograms of the targeted peptides of internal standard proteins. (A, B) unique peptides of histone H4: DNIQGITKPAIR and ISGLIYEETR. (C) unique peptide of prelamin-A/C: NSNLVGAAHEELQQSR. (D) unique peptide of vimentin: TYSLGSALRPSTSR. (E, F) unique peptides of vinculin: AVAGNISDPGLQK and SLGEISALTSK.

UniProt accession	Protein name	Coverage [%]	Sequest HT Score	Peptides	Unique Peptides	Razor Peptides	Normalized Abundance Case1	Normalized Abundance Case2	Normalized Abundance Case3	Normalized Abundance Case4	Normalized Abundance Case5	Normalized Abundance Case6	Normalized Abundance Case7	Normalized Abundance Case8	Normalized Abundance Case9	Normalized Abundance Case10
P02649	Apolipoprotein E	88	3405	38	34	5	70103.6	36481.0	74041.5	61826.8	80382.2	113988.3	58974.8	7505.1	6899.9	67004.5
P04004	Vitronectin	41	2303	17	12	5	11941.7	12608.8	44974.3	27374.4	52827.3	21347.2	39981.0	13637.0	2717.1	11745.6
P02743	Serum amyloid P-component	40	1586	13	13	0	17734.6	16517.8	55684.8	24217.5	6281.8	15395.2	8656.0	17616.7	2786.3	26733.9
P06727	Apolipoprotein A-IV	79	1900	42	40	2	9697.7	10656.1	50047.4	8522.4	30641.2	2982.1	14449.5	13009.9	2017.1	3671.0
P10909	Clusterin	37	1641	20	20	0	10716.1	17174.1	15940.2	5845.4	29828.0	22977.8	14380.4	6701.7	2218.3	5437.2
P01024	Complement C3	63	3960	88	77	11	7660.5	6839.5	9623.7	10643.0	11828.6	10315.9	5797.7	4763.0	4286.3	46288.5
P0DOY2	Immunoglobulin lambda constant 2	70	669	7	3	1	12938.4	12091.3	13431.6	6656.9	271.8	853.5	20705.3	4972.9	2139.3	2116.4
P01834	Immunoglobulin kappa constant	80	887	7	2	0	2121.3	2063.3	463.8	1092.2	16348.1	29887.9	12247.6	353.4	381.9	871.8
P0DOX5	Immunoglobulin gamma-1 heavy chain	45	1825	17	6	12	6036.2	3906.6	3785.4	2144.3	791.1	4104.0	19542.6	4512.9	5033.5	4799.7
P01009	Alpha-1-antitrypsin	65	1092	28	28	0	18271.0	4175.7	6914.4	3011.7	2562.7	4541.1	2030.5	3423.0	5324.5	3269.2
P0C0L5	Complement C4-B	46	2121	62	6	57	2632.8	3939.8	1812.9	7782.4	4574.1	2692.3	2563.4	3243.8	1574.0	19953.8
P02748	Complement component C9	38	924	20	17	2	1090.2	3737.9	4442.0	3972.2	7509.1	7918.8	6656.3	3905.6	1297.1	6889.4
A0A075B6K4	Immunoglobulin lambda variable 3-10	19	16	2	2	0	87.2	73.8	43433.0	70.0	51.7	62.4	83.7	93.9	2884.6	140.9
Q99715	Collagen alpha-1(XII) chain	46	1391	106	106	0	6381.2	5168.8	4511.5	4638.8	2776.0	4138.7	2328.1	2175.7	4178.0	3053.2
P35749	Myosin-11	53	2517	116	2	98	3453.1	9019.8	5233.0	1596.1	1542.9	2383.8	7586.4	4716.8	1295.2	646.5
Q01955	Collagen alpha-3(IV) chain	5	206	6	6	0	3077.0	1004.6	17511.3	1478.4	602.7	308.6	396.0	3923.4	796.5	801.9
O75891	Cytosolic 10-formyltetrahydrofolate dehydrogenase	31	96	22	22	0	5541.5	2197.7	1194.9	843.8	1003.7	2116.1	1394.0	2061.1	3275.7	920.7
P40939	Trifunctional enzyme subunit alpha, mitochondrial	46	536	29	29	0	3385.4	2242.7	1378.2	887.0	1536.6	1771.8	1599.8	2164.1	4630.7	884.3
Q05707	Collagen alpha-1(XIV) chain	40	518	49	49	0	2846.5	1290.9	1210.9	1124.6	935.7	6799.7	900.8	622.5	1646.3	837.8
P0DOX7	Immunoglobulin kappa light chain	54	550	8	3	5	1388.4	1484.6	316.0	764.2	1967.6	4124.4	6617.0	315.2	234.6	845.0
P01871	Immunoglobulin heavy constant mu	31	621	15	15	0	1797.3	1220.8	256.8	4480.2	1612.8	884.3	451.3	554.0	276.6	5265.7
Q9Y6C2	EMILIN-1	28	469	22	22	0	1654.5	1500.7	1764.2	1623.3	2155.1	2468.4	1383.4	1575.6	853.5	1492.6
Q9BRA2	Thioredoxin domain-containing protein 17	27	48	3	3	0	380.6	1177.0	4948.7	1235.0	3621.9	344.2	3068.1	741.3	87.1	165.4
P19827	Inter-alpha-trypsin inhibitor heavy chain H1	22	180	15	13	2	256.2	217.1	178.5	183.3	259.3	195.7	13764.8	164.2	118.2	324.2
P08603	Complement factor H	14	300	17	14	4	563.6	815.1	1673.2	2200.0	1189.2	587.1	374.8	493.6	399.5	5963.7
P28332	Alcohol dehydrogenase 6	31	46	8	8	0	3243.8	974.2	2021.4	742.1	1199.4	657.3	992.5	963.0	2552.0	873.8
P16885	1-phosphatidylinositol 4,5-bisphosphate phosphodiesterase gamma-2	15	23	17	17	0	63.4	102.9	85.5	213.5	49.4	72.2	13018.5	55.1	80.2	39.4
P04003	C4b-binding protein alpha chain	18	335	11	11	0	367.1	1499.0	719.8	3000.3	1441.3	261.8	237.5	1300.5	280.7	3863.3
Q02252	Methylmalonate-semialdehyde dehydrogenase [acylating], mitochondrial	43	530	22	22	0	1749.0	567.1	376.2	221.9	154.6	1013.5	342.1	968.1	7177.4	392.7

Supplemental Table 1: List of significantly high normalized abundance proteins in 10 discovery cohort cases and 10 controls microdissected from renal tissues in parallel.

Abundance Ratio Case1	Abundance Ratio Case2	Abundance Ratio Case3	Abundance Ratio Case4	Abundance Ratio Case5	Abundance Ratio Case6	Abundance Ratio Case7	Abundance Ratio Case8	Abundance Ratio Case9	Abundance Ratio Case10	Normalized Abundance Control1	Normalized Abundance Control2	Normalized Abundance Control3	Normalized Abundance Control4	Normalized Abundance Control5	Normalized Abundance Control6	Normalized Abundance Control7	Normalized Abundance Control8	Normalized Abundance Control9	Normalized Abundance Control10
47.2	24.6	32.8	60.3	81.6	59.2	58.9	5.2	7.1	66.1	1484.5	1482.1	2259.7	1025.3	985.1	1924.3	1001.5	1442.7	978.3	1014.1
12.9	13.1	61.4	33.5	47.6	22.3	22.9	8.9	2.3	7.1	922.9	960.6	732.9	817.7	1110.6	956.8	1744.1	1539.5	1201.3	1646.9
7.4	7.5	78.5	11.7	3.9	6.5	5.1	7.9	1.2	11.8	2404.4	2188.6	709.5	2061.1	1608.0	2371.0	1709.5	2228.8	2254.9	2274.1
10.5	12.8	35.3	10.3	28.4	2.7	16.8	12.5	2.2	4.5	922.5	834.1	1418.9	828.4	1079.1	1119.2	861.9	1042.8	904.7	824.5
8.5	14.4	7.7	5.1	29.2	11.6	16.9	7.8	2.2	5.1	1255.7	1194.3	2066.3	1137.0	1023.2	1982.9	848.6	859.4	1024.8	1058.7
1.5	1.4	1.6	2.2	2.7	2.0	1.6	1.0	0.9	10.7	4948.6	4974.0	5897.8	4913.1	4307.3	5262.5	3553.6	4934.7	4698.5	4322.9
32.4	27.1	101.5	17.4	0.4	2.4	80.4	16.1	6.7	7.4	399.5	446.8	132.4	381.7	665.8	353.1	257.6	307.9	319.3	286.9
3.2	3.2	8.4	1.4	19.8	39.4	13.2	0.4	0.4	1.1	668.6	635.7	55.4	791.7	826.2	758.9	930.4	929.4	1032.8	808.8
2.6	1.2	1.7	0.8	0.3	1.7	10.8	2.2	2.8	2.7	2279.8	3165.0	2245.4	2548.9	2864.0	2428.7	1804.4	2040.8	1802.7	1795.1
13.8	2.6	5.9	1.9	1.4	3.0	2.2	2.7	4.0	2.5	1326.7	1579.0	1165.9	1598.4	1798.3	1510.4	926.8	1276.2	1326.9	1323.2
1.2	1.8	0.6	3.2	2.4	1.3	1.5	1.9	1.0	12.8	2119.2	2229.1	3236.2	2405.5	1915.7	2025.2	1755.1	1737.4	1633.8	1557.3
0.8	3.2	2.4	4.6	6.7	6.3	8.8	5.1	2.9	12.1	1326.8	1155.3	1858.5	862.4	1119.7	1255.8	756.5	762.3	452.5	569.9
3.6	1.4	2396.1	2.2	0.9	1.9	2.9	4.3	83.7	6.3	24.1	53.5	18.1	31.9	57.4	32.5	28.8	21.7	34.4	22.3
8.6	7.4	2.5	8.1	4.1	5.5	4.5	3.3	6.2	5.1	738.5	695.1	1787.4	571.4	679.7	759.0	521.1	659.6	672.9	604.4
3.5	15.8	2.7	2.2	1.6	2.7	13.5	6.0	2.0	1.1	988.4	572.6	1954.1	740.3	947.9	893.3	560.8	788.0	638.3	568.9
1.7	0.7	195.0	0.9	0.4	0.3	0.4	3.9	0.6	0.7	1785.7	1542.4	89.8	1711.6	1374.4	1233.9	951.0	1004.1	1298.5	1144.7
9.1	5.3	1.7	1.9	2.4	4.2	4.5	4.4	6.7	2.1	606.3	415.1	701.1	454.3	422.9	506.4	312.6	464.8	486.4	436.2
5.4	5.6	0.8	1.7	2.3	3.5	5.1	5.7	9.2	2.4	626.9	399.3	1726.9	526.0	662.2	506.2	314.7	379.8	501.4	364.0
8.9	2.2	0.7	2.3	1.5	9.1	1.1	0.9	3.1	1.6	319.4	584.4	1657.5	485.7	628.4	744.1	789.8	656.7	532.2	524.8
2.4	2.3	0.5	1.3	2.7	6.5	13.9	0.5	0.4	1.3	587.6	647.2	579.1	579.5	720.9	633.6	477.4	622.1	605.3	668.8
2.4	1.9	0.4	5.6	1.6	1.1	0.7	0.7	0.3	7.1	759.0	640.6	619.7	796.6	980.2	835.1	684.6	798.4	795.8	746.2
5.0	4.7	5.4	7.1	9.1	7.4	5.3	3.4	1.8	2.7	332.7	321.7	326.1	229.8	237.3	333.1	259.4	459.7	475.3	547.0
10.3	25.7	131.3	19.3	67.6	3.8	47.6	9.2	1.0	1.7	36.8	45.8	37.7	63.9	53.5	89.4	64.5	80.5	88.1	96.7
1.4	2.0	0.1	1.2	1.4	1.2	96.9	1.0	0.7	2.0	181.4	108.0	1540.4	153.6	181.7	168.8	142.0	167.8	178.5	163.3
2.2	2.9	4.6	8.2	4.3	1.9	1.9	2.3	1.5	20.6	258.5	283.8	361.2	269.0	277.3	303.1	198.9	212.6	269.2	289.8
9.2	5.3	9.1	3.8	6.0	2.7	6.1	4.8	20.6	6.2	353.8	183.2	222.5	195.7	201.6	245.7	162.9	202.7	124.1	141.6
2.4	3.1	0.2	14.9	1.4	3.8	325.2	1.0	1.1	1.5	26.8	33.2	352.1	14.4	36.6	19.1	40.0	53.0	72.0	26.4
1.4	5.9	2.8	19.4	6.7	1.5	1.7	7.7	1.5	44.2	270.4	255.3	253.7	154.4	214.4	177.5	136.9	168.8	186.6	87.3
4.2	1.9	1.1	0.4	0.3	1.6	0.9	1.8	15.6	0.8	417.1	302.9	344.6	604.6	496.8	632.8	380.8	541.1	458.7	499.6

UniProt accession	Protein name	Coverage [%]	Sequest HT Score	Peptides	Unique Peptides	Razor Peptides	Average Normalized Abundance samples	Average Normalized Abundance controls	Average Abundance Ratio sample/control	Normalized Abundance CV%	Normalized Abundance Case1	Normalized Abundance Case2	Normalized Abundance Case3	Normalized Abundance Case4	Normalized Abundance Case5	Normalized Abundance Case6	Normalized Abundance Case7	Normalized Abundance Case8	Normalized Abundance Case9	Normalized Abundance Case10
P62805	Histone H4	68	3585.2	14	14	0	13096.7	12985.9	1.0	20.4	12804.1	15265.0	15416.8	14162.5	14200.2	17291.0	6679.0	14297.7	12805.1	8045.9
P02545	Prelamin-A/C	65	3061.7	52	2	55	5326.9	6563.8	0.8	21.3	4556.4	7466.4	4452.5	7379.0	5700.7	4067.9	4811.3	6841.2	3462.3	4531.5
P12111	Collagen alpha-3(VI) chain	50	7492.6	132	20	116	12356.6	12436.9	1.0	22.1	17282.2	11585.5	15006.0	10101.2	11292.5	18348.3	8100.4	8677.7	8332.8	14839.3
P68363	Tubulin alpha-1B chain	47	1925.3	20	0	23	4587.2	6216.1	0.7	26.3	4749.8	4974.8	3379.8	5223.3	4067.6	6721.6	1852.3	5485.5	6180.3	3237.2
P08572	Collagen alpha-2(IV) chain	10	1709.2	11	11	0	4230.0	2944.4	1.4	27.3	4937.0	5269.4	3717.8	5247.3	3575.8	5244.4	3026.3	2466.5	4783.2	4032.2
Q9Y490	Talin-1	59	4354.5	117	117	0	4752.1	6554.8	0.7	27.7	4791.2	6758.8	2181.9	6686.5	5443.1	3961.3	2627.4	5512.4	4113.0	5445.9
P48681	Nestin	55	2369.5	70	70	0	4543.8	4920.3	0.9	28.2	8658.1	6072.5	3785.4	5099.3	3902.5	2693.3	2105.2	4251.8	5003.2	3866.6
P08670	Vimentin	95	8847.7	65	57	8	31351.1	43219.1	0.7	28.3	23777.7	38383.4	20751.2	47035.9	44731.6	24383.1	32641.6	32473.2	13855.9	35477.6
O15230	Laminin subunit alpha-5	34	5843.9	95	94	1	10231.7	14332.9	0.7	28.9	7487.1	11476.0	8673.4	13553.4	11498.3	8732.1	6792.1	16613.4	5842.3	11648.6
P11047	Laminin subunit gamma-1	28	2311.2	47	47	0	4032.7	5947.5	0.7	30.1	3187.8	5449.8	2817.3	6205.3	4619.5	3068.3	2193.8	5607.4	2468.7	4708.9
P21333	Filamin-A	53	4740.1	104	93	12	8318.1	6918.2	1.2	30.1	8752.4	14301.2	7681.6	7173.1	8504.4	7160.0	12334.2	7499.7	4076.7	5697.7
P55268	Laminin subunit beta-2	34	4788.2	56	56	0	7266.7	10024.9	0.7	30.4	5816.8	10002.3	5079.0	11908.4	8398.1	4206.2	5485.0	8235.4	4648.1	8887.7
P60709	Actin, cytoplasmic 1	84	8665.6	32	11	26	42371.6	68770.6	0.6	30.7	37987.7	64901.3	29872.5	58770.3	39449.8	32196.1	31202.2	56138.2	34312.1	38886.3
P84243	Histone H3.3	39	319.1	10	2	8	7886.7	8546.5	0.9	31.2	6393.9	8212.3	8076.9	7589.9	7422.6	8772.8	4458.9	14571.4	7615.3	5753.2
Q16777	Histone H2A type 2-C	59	647.7	9	3	6	11538.9	11025.6	1.0	31.6	9656.9	11343.9	13519.4	9425.7	12199.6	18617.6	6361.5	13755.6	11393.0	9115.7
P18206	Vinculin	66	3090.9	65	65	0	4244.0	5767.8	0.7	31.7	2627.2	5943.2	2975.5	4626.8	4242.4	2590.1	4275.5	5302.2	2629.4	7227.7
P35579	Myosin-9	58	9136.8	140	45	124	13155.1	23759.8	0.6	34.8	11905.1	16490.8	9908.2	17763.2	12986.4	11260.6	8970.9	20413.3	9187.2	12664.8
P02751	Fibronectin	41	2989.6	65	65	0	4971.1	4466.3	1.1	34.9	4433.4	6649.4	2021.5	7397.6	7228.4	3263.5	4140.6	4091.5	1978.4	8506.7
P98160	Basement membrane-specific heparan sulfate proteoglycan core protein	34	6909.9	103	103	0	12556.0	7535.8	1.7	35.2	14684.8	16845.7	8354.6	9823.2	11879.1	17067.3	15605.4	8444.8	10046.1	12809.0
Q16778	Histone H2B type 2-E	67	1279.0	15	3	14	7624.8	8892.7	0.9	38.0	5927.9	8146.2	6101.6	7097.2	6116.0	9181.3	4114.6	15614.5	7758.2	6190.6
P07355	Annexin A2	63	3205.6	29	29	0	3843.7	6940.7	0.6	35.5	3548.4	5180.3	2819.0	4981.7	3412.4	3264.8	2183.2	5868.1	1800.3	5378.7
P68371	Tubulin beta-4B chain	60	2576.0	22	1	29	3719.9	4753.4	0.8	21.6	4316.7	3939.2	2663.7	4351.5	3510.4	4531.1	2031.8	3923.0	5193.7	2737.8
P39060	Collagen alpha-1(XVIII) chain	17	1215.9	23	23	0	3366.6	2039.7	1.7	38.5	4797.4	2885.5	2748.1	3173.1	3354.8	5664.8	2315.3	2977.6	3141.9	2607.1
P62987	Ubiquitin-60S ribosomal protein L40	56	857.3	10	10	0	3288.3	4301.8	0.8	28.9	3298.3	3588.8	3284.9	3297.9	3163.8	3428.3	2392.1	4672.6	3173.2	2582.9
Q09666	Neuroblast differentiation-associated protein AHNAK	66	3473.2	220	220	0	3230.4	6222.8	0.5	37.6	2886.8	4395.2	2982.2	3392.3	2987.3	3211.7	2795.9	3263.1	2927.6	3461.9
P37802	Transgelin-2	64	854.4	11	11	0	3222.8	2338.1	1.4	30.9	3652.2	5123.8	2639.0	4572.1	2370.5	2960.6	2273.1	2206.2	2883.7	3546.5
P12109	Collagen alpha-1(VI) chain	30	2276.5	24	24	0	3142.8	4082.5	0.8	25.7	4301.2	2838.2	4239.6	2815.3	2693.6	4304.0	1721.5	2650.1	2046.7	3817.8
P04406	Glyceraldehyde-3-phosphate dehydrogenase	77	1235.2	23	22	2	3131.4	4475.2	0.7	34.6	3905.7	3512.8	2495.1	3238.7	2374.9	3140.4	1540.3	3980.5	4963.1	2162.5

Supplemental Table 2: List of potential internal standard proteins identified in 10 discovery cohort cases by high normalized protein abundance and non-significant abundance ratio to 10 controls from microdissected renal tissues.

Abundance Ratio Case1	Abundance Ratio Case2	Abundance Ratio Case3	Abundance Ratio Case4	Abundance Ratio Case5	Abundance Ratio Case6	Abundance Ratio Case7	Abundance Ratio Case8	Abundance Ratio Case9	Abundance Ratio Case10	Normalized Abundance control1	Normalized Abundance control2	Normalized Abundance control3	Normalized Abundance control4	Normalized Abundance control5	Normalized Abundance control6	Normalized Abundance control7	Normalized Abundance control8	Normalized Abundance control9	Normalized Abundance control10
0.7	1.4	1.1	1.2	1.3	1.2	0.6	1.2	0.9	0.7	17216.4	11220.6	13889.7	12271.9	11202.4	14795.4	11127.0	11776.0	14106.3	12253.8
0.6	1.1	0.8	1.1	0.9	0.6	0.9	1.0	0.5	0.6	7289.0	6955.8	5409.8	6720.1	6294.0	6385.8	5474.1	6641.4	7070.2	7397.5
1.3	0.8	1.4	0.8	1.0	1.5	0.7	0.7	0.6	1.1	13669.7	13874.0	10849.9	12870.3	10758.9	12435.0	10854.4	11945.4	13740.7	13370.5
0.8	0.8	0.8	0.8	0.7	1.0	0.3	0.8	0.9	0.6	6137.8	6535.5	4369.9	6469.5	6249.0	6743.3	5881.5	6790.0	7225.3	5759.2
1.7	1.7	1.1	1.8	1.4	2.0	0.9	0.8	1.9	1.3	2822.4	3188.1	3264.5	2965.3	2554.3	2601.7	3202.2	3138.2	2576.8	3130.6
0.6	0.8	0.5	0.9	0.8	0.6	0.4	0.8	0.6	0.9	7734.3	8134.1	4565.0	7193.7	6673.7	6126.7	5934.1	6565.0	6376.7	6245.1
1.7	1.1	0.7	1.0	0.8	0.6	0.5	0.9	1.1	0.8	5004.7	5666.5	5704.6	5279.5	4789.3	4390.3	4143.8	4525.7	4618.4	5080.0
0.5	0.7	0.6	1.0	1.1	0.6	0.9	0.9	0.3	0.7	50585.9	53630.7	35684.3	47409.3	41745.0	39389.8	35474.3	35924.2	44646.2	47701.6
0.5	0.8	0.8	0.9	0.6	0.6	0.6	1.2	0.4	0.8	15698.5	15178.4	10746.3	14741.0	19866.7	14189.8	11414.3	13843.1	13466.2	14184.2
0.5	0.8	0.4	0.9	0.7	0.6	0.5	1.1	0.4	0.9	6863.4	6698.9	6475.3	6760.6	6412.4	5305.6	4728.0	5096.3	5625.0	5509.2
1.3	1.6	1.6	0.9	1.3	1.1	2.1	1.1	0.6	0.8	6960.5	8671.4	4948.8	7665.1	6356.0	6674.5	5991.4	7062.9	7345.6	7505.7
0.5	0.9	1.0	1.1	0.9	0.4	0.6	0.8	0.4	0.9	11788.5	11424.5	4962.3	10688.5	9637.4	10482.1	9551.9	10501.1	10872.3	10340.4
0.5	0.9	0.5	0.9	0.6	0.5	0.6	0.9	0.4	0.5	77560.6	72213.5	61661.0	68110.6	69271.9	60460.0	56360.3	65325.9	81918.1	74824.5
0.5	1.1	3.1	0.8	0.6	1.0	0.5	1.7	0.8	0.7	11987.6	7601.6	2617.0	9013.2	11572.6	8845.1	8110.1	8376.7	9075.0	8266.2
0.7	1.1	22.0	0.9	1.1	1.2	0.5	1.2	0.8	0.8	13224.8	10107.3	614.8	10849.1	10965.1	15184.5	12336.3	11757.8	13415.1	11800.8
0.4	0.8	0.8	0.7	0.7	0.5	1.0	1.0	0.5	1.4	7263.0	7890.0	3857.0	6608.3	6037.0	5672.2	4474.6	5298.5	5265.9	5311.1
0.5	0.6	0.5	0.7	0.5	0.4	0.5	1.0	0.4	0.6	23171.4	29855.8	21935.1	26225.9	24586.0	26598.7	19213.3	21360.7	22083.5	22567.2
0.9	1.4	0.6	1.6	1.8	0.8	1.0	0.9	0.4	1.7	4836.1	4850.6	3613.3	4485.7	3965.0	4348.0	4273.4	4700.2	4648.0	4942.4
1.6	1.8	1.1	1.4	1.6	2.6	2.6	1.2	1.3	1.7	9346.5	9386.1	7310.1	7049.6	7296.5	6557.8	6016.1	6929.4	7778.7	7687.3
0.4	0.8	4.2	0.7	0.5	1.0	0.5	2.0	0.7	0.7	13538.4	9833.0	1447.7	9773.7	11323.0	8775.5	7484.3	7755.8	10445.3	8550.1
0.5	0.7	0.4	0.7	0.5	0.5	0.4	0.9	0.3	0.8	7744.0	7675.7	7469.7	6984.0	7071.1	6581.5	5699.3	6237.4	6946.6	6997.9
1.0	0.8	0.6	0.9	0.8	0.9	0.5	0.8	0.9	0.5	4215.1	4639.4	4755.9	4916.0	4308.8	4802.3	4169.3	4797.5	5667.0	5262.4
2.1	1.3	3.3	1.3	1.6	2.4	1.2	1.4	1.5	1.3	2310.1	2220.0	841.3	2486.3	2073.9	2350.7	1977.0	2139.7	2031.6	1966.5
0.9	1.1	0.4	0.9	0.7	0.8	0.7	1.0	0.7	0.7	3782.3	3137.3	7627.2	3711.3	4497.1	4261.7	3348.2	4517.3	4382.1	3753.9
0.5	0.7	0.3	0.5	0.6	0.5	0.5	0.6	0.5	0.5	5592.6	6727.8	9327.9	6243.1	5091.0	5873.9	5149.3	5907.5	5844.8	6470.5
1.6	2.3	1.3	2.5	1.1	1.3	1.0	0.9	0.9	1.3	2320.4	2236.5	2087.9	1820.6	2086.5	2289.7	2213.1	2442.5	3060.2	2824.2
1.1	0.6	1.6	0.7	0.7	1.0	0.5	0.6	0.4	0.8	3901.8	4382.4	2573.3	4301.6	3961.4	4336.5	3589.0	4333.4	4597.3	4847.9
0.8	0.7	2.4	0.6	0.5	0.7	0.4	0.8	0.9	0.4	4848.8	4815.2	1022.3	5564.8	4549.4	4427.5	4263.9	4872.0	5229.7	5158.7

Peptide Sequence	Qvality q-value	UniProt Accessions	Protein Name	Normalized Abundance Case1	Normalized Abundance Case2	Normalized Abundance Case3	Normalized Abundance Case4	Normalized Abundance Case5	Normalized Abundance Case6	Normalized Abundance Case7	Normalized Abundance Case8	Normalized Abundance Case9	Normalized Abundance Case10	Normalized Abundance control1	Normalized Abundance control2	Normalized Abundance control3	Normalized Abundance control4	Normalized Abundance control5	Normalized Abundance control6	Normalized Abundance control7	Normalized Abundance control8	Normalized Abundance control9	Normalized Abundance control10	RT [min]
AATVGSLAGQPLQER	0.0001	P02649	Apolipoprotein E	8346.0	2890.0	6937.3	4897.4	6554.3	12505.8	6257.7	994.2	498.4	5585.3	91.6	78.6	61.1	73.8	57.8	122.4	66.2	117.3	59.6	79.2	69.4
SELEEQLTPVAEETR	0.0001	P02649	Apolipoprotein E	1877.6	1043.1	1523.8	1213.9	2653.3	7468.0	804.4	338.4	453.5	5492.3	47.8	45.2	76.1	48.8	45.3	47.5	22.2	55.1	22.6	31.0	79.1
VEQAVETEPEPELR	0.0001	P02649	Apolipoprotein E	76.6	51.4	0.0	25.5	65.0	84.2	49.8	10.3	11.5	44.9	28.3	7.4	0.0	0.0	23.0	0.0	0.0	6.5	0.0	0.0	54.5
VQAAVGTSAAPVPSDNH	0.0001	P02649	Apolipoprotein E	2465.7	1367.1	4270.3	2633.6	342.5	2082.4	2311.7	247.4	359.1	1702.1	30.9	33.7	0.0	29.3	46.3	35.8	37.7	47.9	30.0	35.5	43.9
WVQTLSEQVQEELLSSQVTQELR	0.0001	P02649	Apolipoprotein E	2661.5	86.1	116.4	1019.9	1106.5	12604.4	324.5	77.1	84.3	1663.5	34.9	37.4	52.6	13.3	23.4	16.8	22.2	22.5	0.0	11.1	117.5
AYSLFSYNTQGR	0.0001	P02743	Serum amyloid P-component	2079.7	1801.2	3691.4	2562.1	731.2	1439.7	719.5	252.1	498.7	2966.7	223.7	179.6	4.8	212.7	37.5	117.9	90.1	214.7	231.5	223.3	84.9
DNELLVYK	0.0003	P02743	Serum amyloid P-component	1762.2	1901.4	8384.6	3010.6	665.6	1390.3	1269.3	248.9	2299.9	3135.4	329.8	305.5	10.5	298.9	131.4	327.6	215.1	136.7	168.3	235.9	69.1
IVLGQEQDSYGGK	0.0001	P02743	Serum amyloid P-component	3595.4	3946.9	12719.1	5657.2	1060.3	2348.4	1864.7	483.5	4285.5	5632.5	416.4	423.8	346.7	398.3	372.0	488.6	201.2	411.7	439.8	460.4	45.8
QGYFVEAQPK	0.0002	P02743	Serum amyloid P-component	3041.2	3132.2	11565.0	5000.3	971.5	2073.6	1930.5	444.6	3332.8	4721.6	370.4	371.0	98.7	332.5	291.4	400.5	327.0	375.7	341.1	390.3	54.8
VGEYSLYIGR	0.0001	P02743	Serum amyloid P-component	2678.4	2162.1	4790.3	3079.7	1035.0	1623.2	1317.3	476.9	443.8	3205.2	417.0	283.2	120.9	216.4	149.7	190.4	327.0	435.9	363.1	394.6	76.9
LAPLAEDVR	0.0002	P06727	Apolipoprotein A-IV	1321.9	1020.3	5254.4	1106.4	5406.6	270.7	2399.8	251.0	1194.3	531.9	49.0	50.1	126.3	54.2	57.6	70.8	58.6	69.5	50.5	55.4	57.5
LEPYADQLR	0.0005	P06727	Apolipoprotein A-IV	1051.9	1615.4	7219.9	1350.2	4260.9	255.0	1859.7	151.1	1782.3	407.2	41.5	39.2	9.9	42.9	53.4	56.1	28.8	46.9	45.4	45.4	52.7
LLPHANEVSQK	0.0002	P06727	Apolipoprotein A-IV	690.8	1121.8	8552.7	744.2	2114.0	157.8	1277.2	160.7	1264.4	319.1	46.0	37.9	4.9	45.4	49.5	49.2	58.4	47.9	38.7	41.4	27.2
LTPYADEFK	0.0003	P06727	Apolipoprotein A-IV	672.4	588.2	3240.8	543.3	2259.9	159.0	1128.4	99.3	522.5	282.5	34.7	45.6	60.5	28.5	23.2	34.1	20.1	26.6	14.7	22.0	67.7
SLAPYAQDTQEK	0.0001	P06727	Apolipoprotein A-IV	618.0	656.2	2629.3	617.9	2179.3	121.4	1182.9	110.5	649.7	284.6	27.1	25.7	0.0	27.2	0.0	45.4	5.5	26.1	19.3	23.0	36.3
FNWYVDGVEVHNAK	0.0001	P0DOX5	Immunoglobulin gamma-1 heavy chain	839.1	47.0	58.4	133.7	16.8	691.4	324.4	315.4	222.7	563.3	106.9	270.6	107.8	121.6	200.8	157.2	268.4	248.2	5.9	228.1	92.2
GPSVFPLAPSSK	0.0001	P0DOX5	Immunoglobulin gamma-1 heavy chain	10.8	3.5	20.8	3.6	0.0	45.1	19.3	20.4	28.3	16.7	7.9	7.7	48.7	7.3	27.6	4.8	6.6	4.0	0.0	0.0	88.0
TTPPVLDSDGSFFLYSK	0.0001	P0DOX5	Immunoglobulin gamma-1 heavy chain	1228.2	778.8	517.8	418.5	59.9	1059.3	3404.7	1206.6	452.3	601.4	420.1	427.8	240.5	472.4	299.2	415.6	365.4	491.4	449.3	400.0	112.3
TVAAPSVFIFPPSDEQLK	0.0001	P01834	Immunoglobulin kappa constant	2186.3	1976.6	481.3	1025.0	18001.2	29791.5	14507.1	400.5	367.5	1030.1	685.2	648.3	43.5	837.4	896.7	770.8	1149.5	996.7	1088.4	877.6	114.5
DSTYSLSSTLTLSK	0.0001	P01834	Immunoglobulin kappa constant	532.2	406.9	66.5	224.9	1378.1	1824.0	2955.4	56.8	75.1	223.5	103.1	158.1	225.6	86.3	112.1	176.6	107.1	240.5	281.2	293.0	92.1
SGTASVVCLLNNFYPR	0.0001	P01834	Immunoglobulin kappa constant	12.6	10.1	0.0	0.0	71.9	295.7	69.8	6.1	4.9	0.0	14.7	13.1	0.0	8.6	0.0	7.4	3.9	7.5	6.9	0.0	119.0
VDNALQSGNSQESVTEQDSK	0.0001	P01834	Immunoglobulin kappa constant	731.7	935.6	228.7	479.4	730.0	1833.3	3939.1	132.1	224.3	667.9	437.9	463.9	361.7	473.5	545.8	396.2	415.8	349.3	270.4	348.5	35.6
ASSLESGVPSR	0.0002	P0DOX7	Immunoglobulin kappa light chain	55.3	73.9	19.0	55.3	19.1	32.3	20.1	13.2	7.2	76.1	18.8	18.1	0.0	19.5	19.7	24.5	24.7	21.8	23.2	19.4	29.9
ASGVPDR	0.0091	A0A087WW87	Immunoglobulin kappa variable 2-40	19.1	18.7	7.0	0.0	25.9	25.7	102.0	11.6	50.6	11.0	1809.6	171.6	212.3	123.9	129.1	11.2	6.6	4.5	2.3	2.1	12.1
ATGIPDR	0.0032	A0A0C4DH25	Immunoglobulin kappa variable 3D-20	236.3	200.8	91.4	168.8	131.0	134.8	175.1	69.7	92.6	217.9	174.5	169.5	336.4	166.2	194.4	96.5	77.7	76.1	75.3	82.8	17.8
AGVETTTPSK	0.0002	P0DOY2	Immunoglobulin lambda constant 2	10939.3	7782.7	4328.3	2672.2	226.8	533.2	15067.6	1942.5	3319.7	2029.1	379.2	375.8	67.5	296.3	515.9	187.3	174.8	170.8	213.5	230.6	14.8
VTHEGSTVEK	0.0016	A0M8Q6	Immunoglobulin lambda constant 7	36.4	29.7	9.2	17.0	0.0	5.0	46.7	0.0	0.0	4.2	13.6	10.8	28.1	0.0	0.0	0.7	0.0	1.5	4.3	3.7	9.6
AAPSVTLFPPSSEELQANK	0.0001	P0DOY2	Immunoglobulin lambda constant 2	3108.7	4830.5	10326.0	4576.5	51.2	328.9	10293.9	316.2	1945.4	531.5	46.9	102.2	0.0	99.0	143.1	174.9	145.9	138.2	122.6	87.3	105.2
SGTSASLAISGLR	0.0002	P01700	Immunoglobulin lambda variable 1-47	87.4	86.0	34.9	66.0	23.0	62.3	3923.8	18.1	10.4	78.7	20.2	20.1	0.0	17.9	26.4	38.3	26.9	27.4	28.4	31.5	77.4
LLIYDNNK	0.0011	P01701	Immunoglobulin lambda variable 1-51	21.9	1.8	12.3	3.0	5.8	11.5	48.0	6.8	0.0	18.8	0.0	8.8	3.3	11.2	2.8	11.5	7.9	0.0	4.8	4.0	58.3
FSGSNSGNTATLTISR	0.0001	P80748	Immunoglobulin lambda variable 3-21	29.0	35.1	10.1	23.2	9.3	17.1	0.0	6.2	4.1	33.4	9.0	6.3	0.0	7.2	9.7	12.6	7.6	11.5	12.4	12.5	54.1
DSERPSGIPER	0.0002	P01718	Immunoglobulin lambda variable 3-27	21.4	518.3	0.0	3.5	5.2	8.0	0.0	0.7	4.0	17.5	0.0	0.0	0.0	0.0	0.0	0.0	0.0	0.0	0.0	1.4	23.8

Supplemental Table 3: List of all identified peptides of amyloidogenic proteins and internal standard proteins in 10 discovery cohort cases and 10 controls microdissected from renal tissues in parallel.

Peptide Sequence	Qvality q-value	UniProt Accessions	Protein Name	Normalized Abundance Case1	Normalized Abundance Case2	Normalized Abundance Case3	Normalized Abundance Case4	Normalized Abundance Case5	Normalized Abundance Case6	Normalized Abundance Case7	Normalized Abundance Case8	Normalized Abundance Case9	Normalized Abundance Case10	Normalized Abundance control1	Normalized Abundance control2	Normalized Abundance control3	Normalized Abundance control4	Normalized Abundance control5	Normalized Abundance control6	Normalized Abundance control7	Normalized Abundance control8	Normalized Abundance control9	Normalized Abundance control10	RT [min]
LLPYIVGVAQR	0.0002	P12111	Collagen alpha-3(VI) chain	33.7	10.8	11.5	10.9	20.5	51.7	0.0	17.4	4.2	23.7	32.2	26.3	0.0	30.3	13.1	18.5	30.1	32.4	31.9	31.6	108.1
LPIGTQQIR	0.0002	P12111	Collagen alpha-3(VI) chain	97.0	41.2	74.8	41.6	35.0	56.0	16.5	43.0	33.6	56.8	63.3	66.3	0.0	73.6	54.7	75.8	58.6	58.2	93.6	87.0	56.0
SDILGHLR	0.0023	P12111	Collagen alpha-3(VI) chain	31.4	20.4	37.1	28.1	1.8	45.3	11.7	27.1	34.7	44.7	42.4	42.5	0.0	45.2	19.1	51.3	37.6	27.0	36.6	31.1	53.8
VGVVQFSDEPR	0.0001	P12111	Collagen alpha-3(VI) chain	101.5	46.1	48.4	51.2	48.0	64.3	18.0	45.7	32.5	50.0	73.3	71.4	6.6	76.7	53.0	68.7	53.6	66.1	70.2	82.8	64.7
YGVVALK	0.0022	P12111	Collagen alpha-3(VI) chain	45.9	17.7	27.6	16.6	33.2	29.8	0.0	20.1	23.5	21.3	0.0	0.0	0.0	43.9	38.2	45.3	46.6	29.8	32.7	32.5	50.2
DAVTYTEHAK	0.0002	P62805	Histone H4	1024.0	1262.4	862.1	1229.8	1585.3	1009.3	499.1	1156.4	1888.2	1033.5	2189.1	1319.5	15.8	1334.3	1550.5	1037.9	1506.8	1016.7	1093.3	1442.7	19.7
DNIQGITKPAIR	0.0001	P62805	Histone H4	5424.5	6919.4	6819.8	6564.5	5471.7	6763.4	3161.1	5880.2	4070.1	4478.0	4326.3	3177.3	35.9	2878.2	1986.5	4047.6	2527.1	3241.1	3297.4	3120.6	57.7
ISGLIYEETR	0.0001	P62805	Histone H4	3158.1	3249.7	4137.5	3854.3	3637.7	4043.9	2130.8	3307.9	3999.9	2015.9	4110.3	2772.4	13088.1	3270.9	3670.3	4908.4	3873.3	3486.9	4370.6	3809.9	64.9
TVTAMDVVYALK	0.0001	P62805	Histone H4	1191.0	1514.4	1173.6	1117.4	732.6	1110.4	542.6	864.6	948.3	650.0	1823.2	634.6	115.9	1217.7	924.5	1131.9	988.8	1383.0	2241.2	1775.9	101.8
TVTAMDVVYALK	0.0001	P62805	Histone H4	7.8	0.0	35.9	0.0	82.4	169.0	0.0	29.7	53.8	15.8	848.6	1185.3	279.5	779.2	95.0	432.6	654.6	93.9	0.0	0.0	112.3
VFLENVIR	0.0003	P62805	Histone H4	2478.8	2376.0	3189.9	2123.9	3892.1	3847.5	1406.4	1893.4	4138.0	1444.2	4456.2	2671.5	451.9	3437.4	3890.7	3323.6	3990.6	3274.4	3654.5	2808.5	92.5
AGALLPAIHEQLR	0.0001	O15230	Laminin subunit alpha-5	265.0	454.2	358.2	549.9	546.7	284.9	97.1	313.7	592.3	488.6	647.0	690.6	0.0	604.0	687.3	982.6	677.2	946.2	602.3	712.0	85.0
AVAAEAQDTATR	0.0001	O15230	Laminin subunit alpha-5	147.3	337.4	116.4	394.4	298.7	110.3	111.1	151.1	431.2	681.1	543.7	492.0	30.8	427.8	527.0	332.8	293.2	294.3	272.9	406.4	15.4
DQASQLLAGTEATLGHAK	0.0001	O15230	Laminin subunit alpha-5	165.3	258.4	109.8	269.4	252.5	76.4	114.5	71.7	149.0	82.5	103.4	131.5	14.0	102.3	169.4	138.3	119.0	154.3	103.3	90.2	95.9
DTLASVFR	0.0010	O15230	Laminin subunit alpha-5	8.9	7.2	21.1	7.5	18.8	28.9	5.1	15.1	52.1	21.3	345.9	297.2	161.8	359.2	27.8	335.5	10.1	16.4	15.8	16.6	79.3
LAASLDGAR	0.0003	O15230	Laminin subunit alpha-5	239.5	489.4	265.7	560.2	481.9	82.5	224.1	233.4	486.1	671.9	328.6	250.2	448.8	212.7	242.0	245.8	384.4	244.3	289.7	262.7	37.4
EAALSTALSEK	0.0002	P02545	Prelamin-A/C	207.0	374.8	162.0	348.5	241.9	199.7	224.0	175.3	166.1	301.8	322.7	360.5	0.0	341.1	255.5	250.8	290.2	270.2	302.8	367.3	52.8
IDSLSAQLSQLQK	0.0001	P02545	Prelamin-A/C	192.1	311.4	184.5	230.6	197.8	192.0	174.4	77.4	229.5	200.3	238.6	307.7	0.0	308.7	255.1	289.9	264.5	336.9	295.4	312.9	91.1
NSNLVGAAHEELQQSR	0.0001	P02545	Prelamin-A/C	259.0	403.6	242.8	431.6	319.5	219.7	228.0	159.9	200.8	326.6	192.4	222.7	65.6	256.5	253.5	370.2	287.3	403.5	360.3	477.5	55.2
ASASGSGAQVGGPISSGSSASSVTVTR	0.0001	P02545	Prelamin-A/C	87.6	152.6	95.0	134.3	91.9	58.7	136.0	53.7	62.9	49.9	67.2	78.1	6.6	47.5	55.2	96.5	65.6	90.2	100.1	112.4	59.4
SVGGSGGGSFGDNLVTR	0.0001	P02545	Prelamin-A/C	125.0	193.1	117.5	223.6	191.1	108.4	218.7	83.4	117.8	142.2	141.9	128.8	86.1	103.5	96.0	130.8	105.1	146.6	148.7	146.1	67.8
EEAENTLQSFR	0.0001	P08670	Vimentin	1094.1	1987.7	1282.3	2821.8	2220.3	1120.7	2227.3	588.1	1547.1	1987.6	2276.9	2677.4	68.0	2470.3	2275.7	2333.1	2095.9	2401.3	2204.5	2653.2	55.4
FADLSEAANR	0.0001	P08670	Vimentin	1061.2	1875.3	1214.6	2585.5	2275.2	933.8	1809.0	556.4	968.4	1889.9	1926.2	2300.9	2501.1	2447.9	1596.7	2376.3	2332.7	2383.4	2223.5	2519.6	45.4
FANYIDK	0.0009	P08670	Vimentin	932.7	1545.8	1260.4	1743.0	2594.2	650.8	1533.1	463.6	1060.8	1708.4	1797.1	2134.4	180.9	1810.6	1592.1	1709.2	2619.2	1468.9	1676.2	1886.9	45.4
QDVDNASLAR	0.0002	P08670	Vimentin	711.6	1502.8	725.7	1851.7	2026.9	735.0	1127.5	570.5	1646.1	2088.6	2628.5	2605.9	75.6	2209.3	2199.0	1389.8	1635.6	1284.5	1105.4	1655.0	25.5
SYVTTSTR	0.0016	P08670	Vimentin	906.6	1549.5	715.5	1537.1	2235.0	764.9	1469.0	777.2	1786.0	2500.8	1940.0	1588.4	14.7	1370.1	1765.3	1238.7	1587.0	1362.9	2101.5	1783.1	17.1
AVAGNISDPGLQK	0.0001	P18206	Vinculin	137.0	282.3	156.7	233.2	121.5	96.0	172.1	107.2	148.4	221.0	242.8	249.2	0.0	230.4	195.2	234.5	334.7	265.3	273.0	268.4	44.9
DPSASPGDAGEQAIR	0.0001	P18206	Vinculin	68.2	209.5	82.2	155.5	110.6	66.1	110.4	71.3	204.1	143.4	203.0	217.4	320.1	205.2	223.7	141.3	158.1	185.1	121.0	162.4	36.8
EAFQPQEPDFPPPPPDLEQLR	0.0001	P18206	Vinculin	28.7	71.5	13.3	58.2	21.8	47.5	41.7	22.2	34.4	63.2	42.4	43.9	84.0	62.3	63.6	41.3	75.4	65.2	77.4	65.6	112.6
SLGEISALTSK	0.0001	P18206	Vinculin	100.2	192.8	124.8	117.0	95.8	96.5	121.5	78.3	122.2	119.3	198.2	203.0	0.0	196.8	134.3	218.5	174.6	205.8	179.3	197.9	72.7
STVEGIQASVK	0.0002	P18206	Vinculin	108.9	258.0	102.9	192.9	85.4	61.8	135.5	77.9	98.4	210.6	142.4	146.4	6.0	150.0	119.3	167.9	260.5	220.0	202.4	208.3	41.4

Protein FDR	Uniprot Accession	Protein Name	Coverage [%]	Sequest HT Score	Peptides	Unique Peptides	Normalized Abundance Case1	Normalized Abundance Case2	Normalized Abundance Case3	Normalized Abundance Case4	Normalized Abundance Case5	Normalized Abundance Case6	Normalized Abundance Case7	Normalized Abundance Case8	Normalized Abundance Case9	Normalized Abundance Case10
High	P02649	Apolipoprotein E	76	1123.46	28	23	1882.80	10262.11	29587.61	25997.42	29088.54	4971.37	48741.54	20605.42	35107.90	41165.92
High	P02743	Serum amyloid P-component	34	582.26	11	11	7507.59	7575.88	2572.16	10277.14	13054.90	8301.26	1828.35	3891.74	7549.07	10845.54
High	P06727	Apolipoprotein A-IV	63	346.78	29	27	3260.03	1377.50	8475.28	3118.56	5452.12	6198.13	2139.27	2644.00	17493.14	1950.76
High	P0DOY2	Immunoglobulin lambda constant 2	42	223.76	3	2	342.90	3529.01	1777.47	3777.93	221.59	1982.78	123.50	4738.08	1034.78	6631.38
High	P01700	Immunoglobulin lambda variable 1-47	15	54.35	2	2	21.83	11.12	16.56	34.70	33.34	33.94	23.19	31.94	25.21	51318.92
High	P0DOX8	Immunoglobulin lambda-1 light chain	30	87.19	6	5	35.93	5531.59	55.17	530.97	33242.77	89.43	90.42	467.71	110.27	147.15
High	P01834	Immunoglobulin kappa constant	50	64.54	5	2	69.94	84.53	88.43	528.81	1046.90	229.50	366.85	451.18	208.48	270.05
High	P0DOX7	Immunoglobulin kappa light chain	30	35.31	5	2	38.58	53.97	75.68	197.71	542.56	160.26	564.20	253.55	74.87	155.25
High	A0A075B6S5	Immunoglobulin kappa variable 1-27	14	2.63	1	1	0.00	0.00	0.00	4.92	0.00	0.00	0.00	0.00	0.00	0.00
High	P01624	Immunoglobulin kappa variable 3-15	26	6.59	2	2	5.86	10.67	13.02	88.87	77.41	25.20	29.89	61.86	52.35	28.19
High	P01619	Immunoglobulin kappa variable 3-20	28	35.74	3	3	48.13	77.98	148.56	182.02	259.28	103.02	108.45	139.23	252.95	98.95
High	A0A0A0MRZ8	Immunoglobulin kappa variable 3D-11	8	9.03	1	1	4.28	4.32	8.18	52.33	46.70	15.63	16.21	27.20	16.66	12.13
High	P01857	Immunoglobulin heavy constant gamma 1	20	231.65	6	4	146.56	3382.67	409.70	1214.45	1559.24	867.90	1389.40	1391.83	488.50	1579.34
High	P01876	Immunoglobulin heavy constant alpha 1	19	75.33	6	3	105.68	80.48	37.77	1655.48	3516.05	325.30	297.57	334.25	358.66	141.09
High	P01877	Immunoglobulin heavy constant alpha 2	12	23.02	4	0	0.00	0.00	0.00	0.00	0.00	0.00	0.00	0.00	0.00	0.00
High	P01859	Immunoglobulin heavy constant gamma 2	12	106.86	4	2	4.23	0.00	0.00	22.13	93.22	7.58	27.71	92.72	3.71	26.24
High	P01861	Immunoglobulin heavy constant gamma 4	16	64.73	3	2	0.00	0.00	0.00	14.61	41.41	0.00	12.33	38.72	0.00	12.18
High	P01871	Immunoglobulin heavy constant mu	31	91.52	13	13	239.01	152.35	347.51	2343.44	680.39	566.22	686.26	635.60	623.89	375.34
High	P01780	Immunoglobulin heavy variable 3-7	23	1.74	3	3	0.00	0.00	0.00	42.28	44.86	0.00	6.60	6.96	3.00	3.90
High	P06331	Immunoglobulin heavy variable 4-34	7	3.90	1	1	4.41	5.30	15.06	42.95	41.64	11.04	21.04	20.79	15.71	20.56

Supplemental Table 4. Untargeted proteomic diagnostic signature for 10 cases of early-stage renal amyloidosis.

Abundance Ratio Case1	Abundance Ratio Case2	Abundance Ratio Case3	Abundance Ratio Case4	Abundance Ratio Case5	Abundance Ratio Case6	Abundance Ratio Case7	Abundance Ratio Case8	Abundance Ratio Case9	Abundance Ratio Case10
1.89	10.33	29.77	26.16	29.27	5.00	49.04	20.73	35.33	41.42
5.57	5.62	1.91	7.62	9.68	6.15	1.36	2.89	5.60	8.04
7.27	3.07	18.91	6.96	12.16	13.83	4.77	5.90	39.03	4.35
2.48	25.55	12.87	27.36	1.60	14.36	0.89	34.31	7.49	48.02
0.09	0.04	0.07	0.14	0.13	0.14	0.09	0.13	0.10	205.84
0.26	40.20	0.40	3.86	241.59	0.65	0.66	3.40	0.80	1.07
1.07	1.29	1.35	8.09	16.01	3.51	5.61	6.90	3.19	4.13
0.08	0.12	0.16	0.43	1.17	0.35	1.22	0.55	0.16	0.34
0.00	0.00	0.00	0.94	0.00	0.00	0.00	0.00	0.00	0.00
0.09	0.16	0.20	1.36	1.19	0.39	0.46	0.95	0.80	0.43
0.09	0.14	0.27	0.33	0.46	0.18	0.19	0.25	0.45	0.18
0.11	0.11	0.21	1.32	1.18	0.40	0.41	0.69	0.42	0.31
0.15	3.52	0.43	1.26	1.62	0.90	1.45	1.45	0.51	1.64
0.14	0.11	0.05	2.23	4.74	0.44	0.40	0.45	0.48	0.19
0.00	0.00	0.00	0.00	0.00	0.00	0.00	0.00	0.00	0.00
0.01	0.00	0.00	0.04	0.17	0.01	0.05	0.17	0.01	0.05
0.00	0.00	0.00	2.50	7.08	0.00	2.11	6.62	0.00	2.08
0.27	0.17	0.40	2.68	0.78	0.65	0.78	0.73	0.71	0.43
0.00	0.00	0.00	2.19	2.32	0.00	0.34	0.36	0.16	0.20
0.16	0.19	0.54	1.55	1.50	0.40	0.76	0.75	0.57	0.74

Protein Name	Peptide Sequence	Case1-1	Case1-2	Case1-3	Case2-1	Case2-2	Case2-3	Case3-1	Case3-2	Case3-3	Case4-1	Case4-2	Case4-3	Case5-1	Case5-2	Case5-3
Apolipoprotein E	AATVGSLAGQPLQER	0.5105	0.3070	0.2868	2.2715	2.7588	2.8940	13.9272	2.2787	2.1778	8.6117	6.6032	6.9327	17.7214	10.3412	13.0340
Apolipoprotein E	SELEEQLTPVAEETR	0.1317	0.1078	0.0898	1.1896	2.2193	2.3639	3.9726	0.7719	0.6949	1.8894	1.5483	1.6126	4.0817	2.8111	2.8633
Apolipoprotein E	VEQAVETEPEPELR	0.0003	0.0004	0.0004	0.0075	0.0254	0.0246	0.0308	0.0064	0.0069	0.0141	0.0153	0.0152	0.1191	0.0889	0.0834
Serum amyloid P-component	VGEYSLYIGR	4.1456	1.5855	1.4676	4.6935	1.7229	1.7285	2.2164	0.3350	0.3219	8.3000	4.6716	4.5088	11.3336	9.0871	9.8164
Serum amyloid P-component	IVLGQEQDSYGGK	2.1530	1.2040	1.0699	2.5133	4.3202	4.3198	1.2637	0.5359	0.4845	3.8532	3.1331	3.3214	1.6786	1.1287	1.1081
Serum amyloid P-component	AYSLFSYNTQGR	0.4399	0.0240	0.1923	0.2478	0.2921	1.7758	0.0001	0.0003	0.0305	0.3266	0.0087	3.0629	0.0384	0.3941	0.2903
Apolipoprotein A-IV	LAPLAEDVR	0.5473	0.3128	0.3027	0.2363	0.2302	0.2354	2.9481	0.6729	0.6704	0.8376	0.5948	0.6309	10.3746	9.4586	10.4537
Apolipoprotein A-IV	LEPYADQLR	0.4272	0.3423	0.3019	0.2360	0.1971	0.2158	2.0555	0.5941	0.5971	0.9521	0.8448	0.8532	13.1811	12.0160	12.2800
Apolipoprotein A-IV	SLAPYAQDTQEK	0.2360	0.1641	0.1453	0.1116	0.1156	0.1252	1.3235	0.2879	0.2936	0.3233	0.2725	0.2808	1.3850	1.9687	1.9964
Immunoglobulin lambda light chain	SGTSASLAISGLR	0.0084	0.0031	0.0026	0.0245	0.0139	0.0138	0.1644	0.0221	0.0225	0.1259	0.1115	0.1175	0.1159	0.0636	0.0645
Immunoglobulin lambda light chain	AAPSVTLFPPSSEELQANK	0.6991	0.4601	0.4226	7.7564	7.4940	9.2687	4.3713	0.6886	0.7580	21.2517	10.8685	10.7746	5.6224	4.8463	5.6462
Immunoglobulin lambda light chain	AGVETTTPSK	0.1584	0.1606	0.1520	2.2451	3.0237	3.2771	1.8601	0.2528	0.2609	0.9832	0.8470	0.8669	0.9911	0.6877	0.6810
Immunoglobulin kappa light chain	TVAAPSVFIFPPSDEQLK	0.0445	0.0279	0.0265	0.0684	0.0554	0.0818	0.2256	0.0653	0.2394	0.6222	0.3424	0.4508	0.2878	0.2109	0.3376
Immunoglobulin gamma-1 heavy chain	GPSVFPLAPSSK	0.0967	0.0744	0.0712	2.7987	3.2930	3.7512	0.5531	0.1713	0.2109	0.9828	0.7818	0.7997	0.3364	0.2774	0.2311
Immunoglobulin gamma-1 heavy chain	TTPPVLDSDGSFFLYSK	0.0087	0.0058	0.0054	0.3865	0.2348	0.1486	0.0721	0.0185	0.0281	0.0718	0.0406	0.0533	0.0466	0.0255	0.0290

Supplemental Table 5. PRM-based targeted proteomic diagnostic signature for 10 cases early-stage renal amyloidosis.

Case6-1	Case6-2	Case6-3	Case7-1	Case7-2	Case7-3	Case8-1	Case8-2	Case8-3	Case9-1	Case9-2	Case9-3	Case10-1	Case10-2	Case10-3	Cut Off Value
0.4240	0.4314	0.3801	9.4110	10.9886	11.2893	3.6346	3.5794	3.6970	5.3171	5.1717	5.3736	7.5853	5.0966	3.9116	0.4639
0.1523	0.1991	0.1847	4.1407	4.1937	3.5817	0.7885	0.8961	0.8736	1.5638	1.4298	1.5241	2.8786	1.8379	1.5608	0.0811
0.0028	0.0009	0.0014	0.0327	0.0364	0.0319	0.0046	0.0385	0.0401	0.0190	0.0141	0.0143	0.0178	0.0186	0.0197	0.0012
1.9221	1.1815	1.0543	0.6383	0.5813	0.3449	1.1407	0.8032	0.7522	4.2448	3.6213	3.5176	3.6006	3.2788	2.9864	0.5511
1.0537	1.9884	1.9875	0.4079	0.5236	0.5007	0.6535	0.7185	0.7368	1.9397	2.5961	2.7267	1.7226	1.4985	1.4587	0.3733
0.1967	0.1202	0.0484	0.0198	0.0907	0.0518	0.0652	0.0047	0.1365	0.1389	1.0966	2.3882	0.2379	0.0890	0.5601	0.7298
0.2851	0.4617	0.4881	0.0581	0.0689	0.0674	0.2052	0.1806	0.1870	0.5723	0.6059	0.6296	0.1545	0.4132	0.2584	0.6155
1.0645	1.9958	1.9844	0.0705	0.0831	0.0811	0.2942	0.3400	0.3386	0.5130	0.5122	0.5448	0.2679	0.5494	0.5256	0.4947
0.0886	0.1808	0.1778	0.0199	0.0293	0.0290	0.0960	0.0914	0.0877	0.2107	0.2447	0.2520	0.0590	0.1056	0.0984	0.2480
0.0178	0.0223	0.0210	0.0269	0.8885	0.8214	0.0390	0.0338	0.0343	0.0439	0.0802	0.0815	81.8002	46.0767	41.9785	0.2104
2.2365	4.2235	3.9570	0.2196	0.3760	0.4140	5.7973	4.6539	4.3113	0.3085	0.3797	0.4386	7.7427	4.5517	4.1182	0.4594
0.3472	0.5899	0.5827	0.0525	0.0739	0.0855	1.2158	1.0507	1.0038	0.0154	0.0116	0.0151	1.6769	1.0734	0.9873	0.0242
0.1245	0.1157	0.2063	0.1509	0.3812	0.2422	0.2208	0.1243	0.1156	0.6707	0.6833	0.6546	0.1500	0.1755	0.1417	0.7567
0.2385	0.4007	0.3887	0.8930	1.0226	1.0450	0.8182	0.2829	0.2513	0.4984	0.6689	0.6863	0.4337	0.3743	0.3643	1.0779
0.0163	0.0264	0.0306	0.0644	0.0786	0.0615	0.0501	0.0163	0.0221	0.0327	0.0503	0.0665	0.0510	0.0410	0.0199	0.1543

Supplemental Table 1: List of significantly high normalized abundance proteins in 10 discovery cohort cases and 10 controls microdissected from renal tissues in parallel. 10 representative renal amyloid and 10 control tissues from formalin-fixed paraffin-embedded biopsy specimens are presented on the x-axis, and the evaluated proteins are presented on the y-axis. Numbers in the yellow boxes indicate a significantly high normalized abundance of matching proteins as amyloidogenic proteins in a sample. The numbers in the green boxes indicate the amyloidogenic protein abundance ratio to controls in a sample. A polipoprotein E, serum amyloid P-component, and apolipoprotein A-IV are amyloid signature proteins (protein FDR < 0.01, Exp. q-value<0.01)

Supplemental Table 2: List of potential internal standard proteins identified in 10 discovery cohort cases by high normalized protein abundance and non-significant abundance ratio to 10 controls from microdissected renal tissues. 10 representative renal amyloid and 10 control tissues from formalin-fixed paraffin-embedded biopsy specimens are presented on the x-axis, and the evaluated proteins are presented on the y-axis. Red font rows indicate screened proteins with easy detectability and stable expression (protein FDR < 0.01, Exp. q-value<0.01, CV%: coefficient of variation).

Supplemental Table 3: List of all identified peptides of amyloidogenic proteins and internal standard proteins in 10 discovery cohort cases and 10 controls microdissected from renal tissues in parallel. 10 representative renal amyloid and 10 control tissues from formalin-fixed paraffin-embedded biopsy specimens are presented on the x-axis, and evaluated peptides are presented on the y-axis (FDR < 0.01).

Supplemental Table 4. Untargeted proteomic diagnostic signature for 10 cases of early-stage renal amyloidosis. Early-stage renal amyloidosis cases based on formalin-fixed paraffin-embedded biopsy specimens are presented on the x-axis, and evaluated proteins are presented on the y-axis. Numbers in the yellow boxes indicate a significantly high normalized abundance of matching amyloid precursor proteins in a sample. Numbers in the green boxes indicate the abundance ratio to control tissues of amyloid precursor protein in a sample. Apolipoprotein E, serum amyloid P-component, and apolipoprotein A IV are amyloid signature proteins. Case2: AHL IgG1-λ; Case4, Case5, Case8, Case10: AL-λ; Case1, Case3, Case6, Case7, Case9: unclassified types.

Supplemental Table 5. PRM-based targeted proteomic diagnostic signature for 10 cases early-stage renal amyloidosis. 10 early-stage renal amyloidosis cases based on formalin-fixed paraffin-embedded biopsy specimens are presented on the x-axis, and evaluated peptides and corresponding proteins are presented on the y-axis. Numbers in the yellow boxes indicate higher targeted protein abundance ratios to internal standard proteins than the cut-off values in a sample. Case2: AHL IgG1-λ; Case1, Case3, Case4, Case5, Case6, Case7, Case8, Case10: AL-λ; Case9: unclassified types.
